# Critically short telomeres derepress retrotransposons to promote genome instability in embryonic stem cells

**DOI:** 10.1038/s41421-023-00538-y

**Published:** 2023-05-02

**Authors:** Nannan Zhao, Guoxing Yin, Chun Liu, Weiyu Zhang, Yang Shen, Dan Wang, Zhenzhen Lin, Jiao Yang, Jian Mao, Renpeng Guo, Yongwang Zhang, Feng Wang, Zhe Liu, Xinyi Lu, Lin Liu

**Affiliations:** 1 State Key Laboratory of Medicinal Chemical Biology, Nankai University, Tianjin, China; 2 Frontiers Science Center for Cell Responses, College of Life Sciences, Nankai University, Tianjin, China; 3 College of Pharmacy, Nankai University, Tianjin, China; 4 Genome Institute of Singapore, Singapore, Singapore; 5 2011 Collaborative Innovation Center of Tianjin for Medical Epigenetics, Tianjin Key Laboratory of Medical Epigenetics, Department of Immunology, Biochemistry and Molecular Biology, School of Basic Medical Sciences, Tianjin Medical University, Tianjin, China; 6 Department of Genetics, School of Basic Medical Sciences, Tianjin Medical University, Tianjin, China; 7 Haihe Laboratory of Cell Ecosystem, Chinese Academy of Medical Sciences & Peking Union Medical College, Tianjin, China; 8 Institute of Translational Medicine, Tianjin Union Medical Center, Nankai University, Tianjin, China

**Keywords:** Embryonic stem cells, Ageing

## Abstract

Telomeres, at the ends of chromosomes, protect chromosomes from fusion and preserve genomic stability. However, the molecular mechanisms underlying telomere attrition-induced genome instability remain to be understood. We systematically analyzed the expression of retrotransposons and performed genomic sequencing of different cell and tissue types with telomeres of varying lengths due to telomerase deficiency. We found that critically short telomeres altered retrotransposon activity to promote genomic instability in mouse embryonic stem cells, as evidenced by elevated numbers of single nucleotide variants, indels and copy number variations (CNVs). Transpositions of retrotransposons such as LINE1 resulting from the short telomeres can also be found in these genomes with elevated number of mutations and CNVs. Retrotransposon activation is linked to increased chromatin accessibility, and reduced heterochromatin abundance correlates with short telomeres. Re-elongation of telomeres upon recovery of telomerase partly represses retrotransposons and heterochromatin accumulation. Together, our findings suggest a potential mechanism by which telomeres maintain genomic stability by suppressing chromatin accessibility and retrotransposon activity.

## Introduction

Telomeres protect chromosome ends to prevent chromosome fusion and instability^1^. It has long been recognized that telomere dysfunction and rearrangement in subtelomeric and telomeric regions can lead to genome instability and eventually cause cancer and other diseases in human^2,3^. Telomeres shorten progressively with age, and this shortening is accompanied by the accumulation of somatic mutations and genome instability^4,5^. Telomeres are also generally shorter in tumor cells than in normal somatic cells^6^. Recently, the mapping of somatic mutations revealed variable mutation rates in different tissues^7^. However, the relationships and mechanisms underlying short or dysfunctional telomere-induced mutations and genomic instability remain elusive.

Endogenous retroviruses (ERVs) constitute ~8%–10% of mammalian genomes^8^. Repression of retrotransposons is critical for the maintenance of genome stability in both somatic cells and embryonic stem cells (ESCs)^9^. Most retrotransposons are silenced to prevent genome instability^10^, but in some cases, retrotransposons are activated. For instance, long terminal repeat (LTR) retrotransposons are highly expressed in the mammalian placenta and trophectoderm^11^. Intriguingly, retrotransposons are activated in a small number of cases during early embryonic development, contributing to pluripotent identity and networks in mouse and human pluripotent stem cells^12–16^. Moreover, retrotransposon MERVL and genes in the 2-cell embryo are sporadically activated in only a small proportion (1%–5%) of ESC cultures^17,18^. In somatic cells, the activation of retrotransposons can drive cancer gene expression and lead to cancer development^19^. They can also be activated during replicative senescence in mouse and human cells^20^. The accumulation of retrotransposon RNAs can induce inflammation and immune responses^21^ and result in cellular senescence^22,23^. Short telomeres can also lead to cell replicative senescence^24–26^. Furthermore, the shortest telomeres, not the average telomere length, is implicated in cell senescence^27^. It is noted that human telomeres are much shorter than mouse telomeres and that mouse somatic cells continue expressing telomerase in adulthood^28^, such that only late-generation telomerase knockout (*Terc*^−/−^) mice exhibit short telomere-associated phenotypes, including accelerated aging, reduced injury repair and predisposition to tumorigenesis^29^. Such mice have been extensively used as study models of telomere biology and function in humans. *Terc* is an essential RNA component of the telomerase complex whose absence affects telomerase activity and causes telomere shortening in diseases^30^ and is important for pluripotent stem cells^31^. Short telomeres in mouse ESCs without telomerase component *Terc* lead to reduced cell proliferation^32^, similar to replicative senescence in human cells. It remains unknown whether telomere length regulates retrotransposons to influence genome stability in mammalian cells.

Herein, by systematically examining the effects of telomere length on the transcriptome and on genome integrity in various cell types following telomere shortening, we investigated the role of telomere length in the maintenance of genome stability by controlling the expression of retrotransposons. Critically short telomeres are associated with retrotransposon activation and their subsequent retrotransposition to promote genomic instability.

## Results

### Telomere attrition in various mouse cell types and tissues lacking telomerase

We first compared telomere lengths by telomere restriction fragment (TRF) analysis of various somatic tissues or cells obtained from the third-generation (G3) *Terc*^−/−^ mice and ESCs (G4 *Terc*^−/−^) derived from blastocysts of intercrosses of G3 *Terc*^−/−^ mice. G1 *Terc*^−/−^ ESCs were derived from blastocysts of intercrosses of *Terc*^+/−^ mice while G3 *Terc*^−/−^ ESCs were derived from blastocysts of intercrosses of G2 *Terc*^−/−^ mice^32^. Telomeres were shortened in all somatic tissues including the tail, testis and liver, as well as in cells in culture such as adipose-derived mesenchymal cells (ASCs), tail tip fibroblasts (TTFs) and ESCs obtained from G3 *Terc*^−/−^ mice, compared with wild-type (WT) mice (Supplementary Fig. S1a, b). Moreover, telomeres were shortened in *Terc*^−/−^ ESCs with an increasing number of generations (G) from G1 to G4 and with increasing cell culture passages (P) from P18 to P42. P18 was considered as an early passage, and P42 was considered as late passage. G3 *Terc*^−/−^ ESCs had shorter telomeres than G1 *Terc*^−/−^ ESCs, but longer telomeres than G4 *Terc*^−/−^ ESCs. Telomere lengths were measured in somatic cells (TTFs and ASCs) at an earlier passage (P3) because our preliminary observations indicated that these cells would undergo senescence in the absence of telomerase. G4 *Terc*^*−/−*^ ESCs had the shortest telomeres among all tissues, whereas WT ESCs had the longest telomeres (Supplementary Fig. S1a, b).

Telomere quantitative fluorescence in situ hybridization (Q-FISH) of chromosome spreads confirmed the occurrence of telomere shortening in *Terc*^*−/−*^ cells (Supplementary Fig. 1c–e). Notably, G4 *Terc*^−/−^ ESCs at both early and late passages exhibited critically short telomeres and telomere loss, as evidenced by telomere signal-free chromosome ends and end-to-end chromosome fusions (Supplementary Fig. S1c–e). G4 *Terc*^−/−^ ESCs at both early and late passages exhibited chromosome fusions (Supplementary Fig. S1f). In contrast, somatic TTFs derived from G3 *Terc*^−/−^ mice showed no obvious telomere loss or chromosome fusion (Supplementary Fig. S1c–e). G4 *Terc*^−/−^ ESCs had the shortest telomeres according to Q-FISH assays (Supplementary Fig. S1c–e), while telomeres were further shortened by passaging of the ESCs (Supplementary Fig. S1c–e). These results also substantiate the notion that telomerase is critical for telomere maintenance in both somatic cells and ESCs. However, mouse ESCs proliferate very rapidly, with cell doubling approximately every 7 h, unlike somatic cells, which may explain the shorter telomeres found in ESCs than in somatic cells lacking telomerase obtained from mice of the same generation.

### Short telomeres alter retrotransposons

**Fig. 1:**
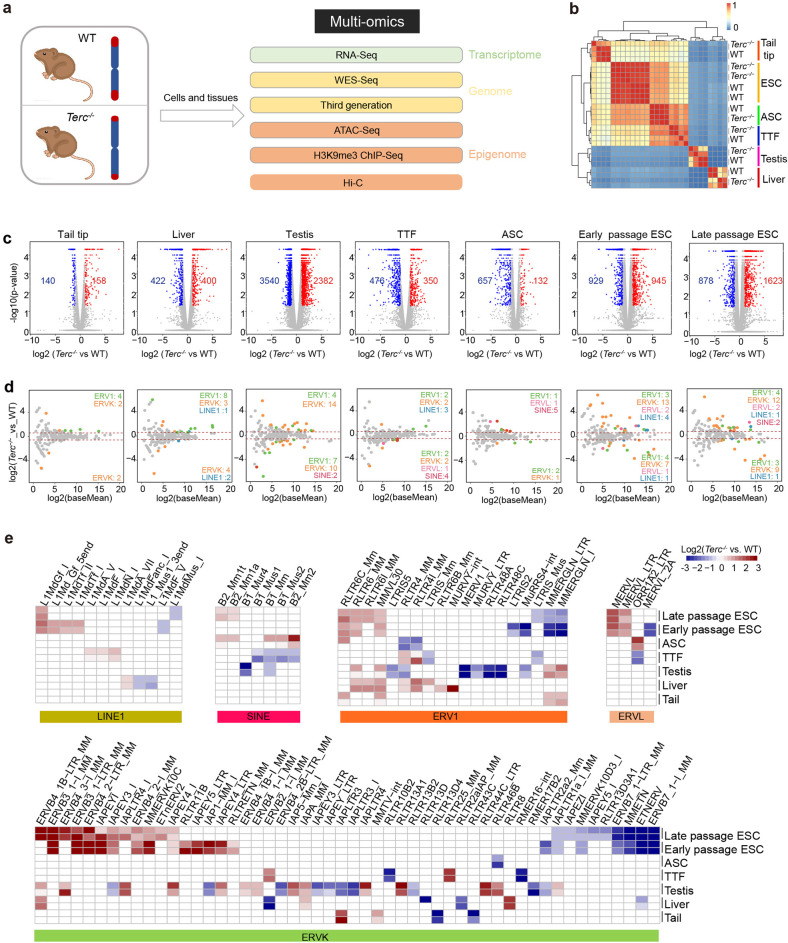
Transcriptome of differentially expressed genes and retrotransposons of various cell types from *Terc*^*−/−*^ and WT mice. **a** Overall experimental design. Several types of cultured cells and tissues from WT and G3 *Terc*^*−/−*^ mice were used for multi-omics analysis, including the transcriptome analysis by RNA-seq, whole-exome sequencing (WES), third-generation whole-genome sequencing, and epigenome analysis using ATAC-seq, ChIP-seq of H3K9me3, and Hi-C sequencing. **b** Heatmap showing the correlation of the expressed genes among the cell types and tissues. ESC embryonic stem cell (P18 for early passage and P42 for late passage), TTF tail-tip fibroblast, ASC adipose-derived mesenchymal cell. The passage number for somatic tissues is P3. The bar color represents the correlation coefficient of the samples. **c** Volcano plot of differentially expressed genes (DEGs) in *Terc*^*−/−*^ and WT cells of various cell types. Red dots, upregulated genes with fold change > 2 and *P* value < 0.05; blue dots, downregulated genes with fold change > 2 and *P* value < 0.05; grey dots, genes without significant change. Early-passage G4 *Terc*^*−/−*^ ESC (P18), late-passage G4 *Terc*^*−/−*^ ESC (P42), G3 *Terc*^*−/−*^ TTF (P3), G3 *Terc*^*−/−*^ ASC (P3), and fresh tissues were collected. **d** MA plot illustrating differentially expressed retrotransposons of the LINE1, ERV1, ERVL and ERVK classes in *Terc*^*−/−*^ and WT cells or tissues. Significantly changed retrotransposons are shown in different colors according to class. **e** Heatmap of all differentially expressed TEs by fold change in short telomere tissues or cells compared with WT tissues or cells. The bar color represents the fold change of the expression of TEs in *Terc*^*−/−*^ vs WT cells; red indicates retrotransposons upregulated in *Terc*^*−/−*^ vs WT cells; blue indicates retrotransposons downregulated in *Terc*^*−/−*^ vs WT cells.

To assess the impact of short telomeres on transcriptome and genomic stability, we performed multi-omics analyses to compare various tissues and cell types between G3 *Terc*^−/−^ mice and WT mice. These included RNA-seq to interrogate the transcriptome, whole exome-seq and third-generation genome sequencing to examine genome stability, high-throughput/resolution chromosome conformation capture (Hi-C) to observe 3D chromatin organization, and ATAC-seq and chromatin immunoprecipitation (ChIP) coupled with high-throughput sequencing (ChIP-seq) of H3K9me3 to identify epigenomic changes (Fig. 1a). We systematically compared the expression of genes and retrotransposons in somatic tissues and cultured cells. The global gene expression profile of ESCs differed from those of other cell types (Fig. 1b). The strongest changes in gene expression among the somatic tissues and cells assessed occurred in *Terc*^−/−^ testes, most likely because the testis contains a variety of cell types (Fig. 1c). *Terc*^−/−^ ESCs displayed more changes in gene expression than somatic cells such as TTFs and ASCs (Fig. 1c). This might be because ESC cultures undergo more cell passages and rapid cell proliferation, which resulted in short telomeres in the absence of telomerase, whereas only three passages were performed for the somatic cells, as explained above.

The expression of retrotransposons was minimally affected in somatic cell TTFs, ASCs and tail tip samples isolated from G3 *Terc*^−/−^ mice compared to WT cells (Fig. 1d). More retrotransposons were activated in liver and testis tissues with short telomeres, which consist of more diverse cell types than other somatic cell types (Fig. 1d). In contrast, more retrotransposons were altered and activated in G4 *Terc*^−/−^ ESCs, derived from blastocysts of G3 *Terc*^−/−^ mice, than in somatic cell types of G3 *Terc*^−/−^ mice (Fig. 1d). Notably, retrotransposons with transposition activity including LINE1 family members were activated most in ESCs (Fig. 1d, e). Among the different cell types, testes and ESCs demonstrated the most changes in retrotransposon expression (Fig. 1d, e). This is probably due to the high proliferation rate of ESCs and spermatogonia stem cells in addition to the presence of more cell types within the testis, which also showed more gene expression changes after telomere shortening (Fig. 1c). Moreover, retrotransposon activation was mainly conserved across passages of ESCs (Fig. 1d). These results suggest that telomere length is a key factor in repressing retrotransposons across different cell types. Furthermore, *Terc*^−/−^ ESCs, with the shortest telomeres, showed activation of retrotransposons from all transposable element (TE) families, whereas only a minority of retrotransposon classes were activated in other tissues or cell types where telomeres were not critically shortened (Fig. 1e). Cells with higher proliferation activity and without telomerase exhibited a more rapid shortening of telomeres. Critically short telomeres in G4 *Terc*^−/−^ ESCs after in vitro culture are associated with retrotransposon derepression (Fig. 1e). In contrast, retrotransposons, which were downregulated in cells with short telomeres, were fewer in number and were expressed at overall low levels in WT ESCs (Fig. 1e and Supplementary Fig. S2a). In addition, unlike the upregulated retrotransposons, the downregulated retrotransposons, such as short interspersed nuclear elements (SINEs), are nonautonomous. We speculate that the downregulation of these retrotransposons could be an indirect effect caused by other alterations in G4 *Terc*^−/−^ ESCs, which remain to be understood. Therefore, our study mainly focused on the upregulated retrotransposons such as LINE1s, which have been known to retrotranspose^33,34^.

**Fig. 2:**
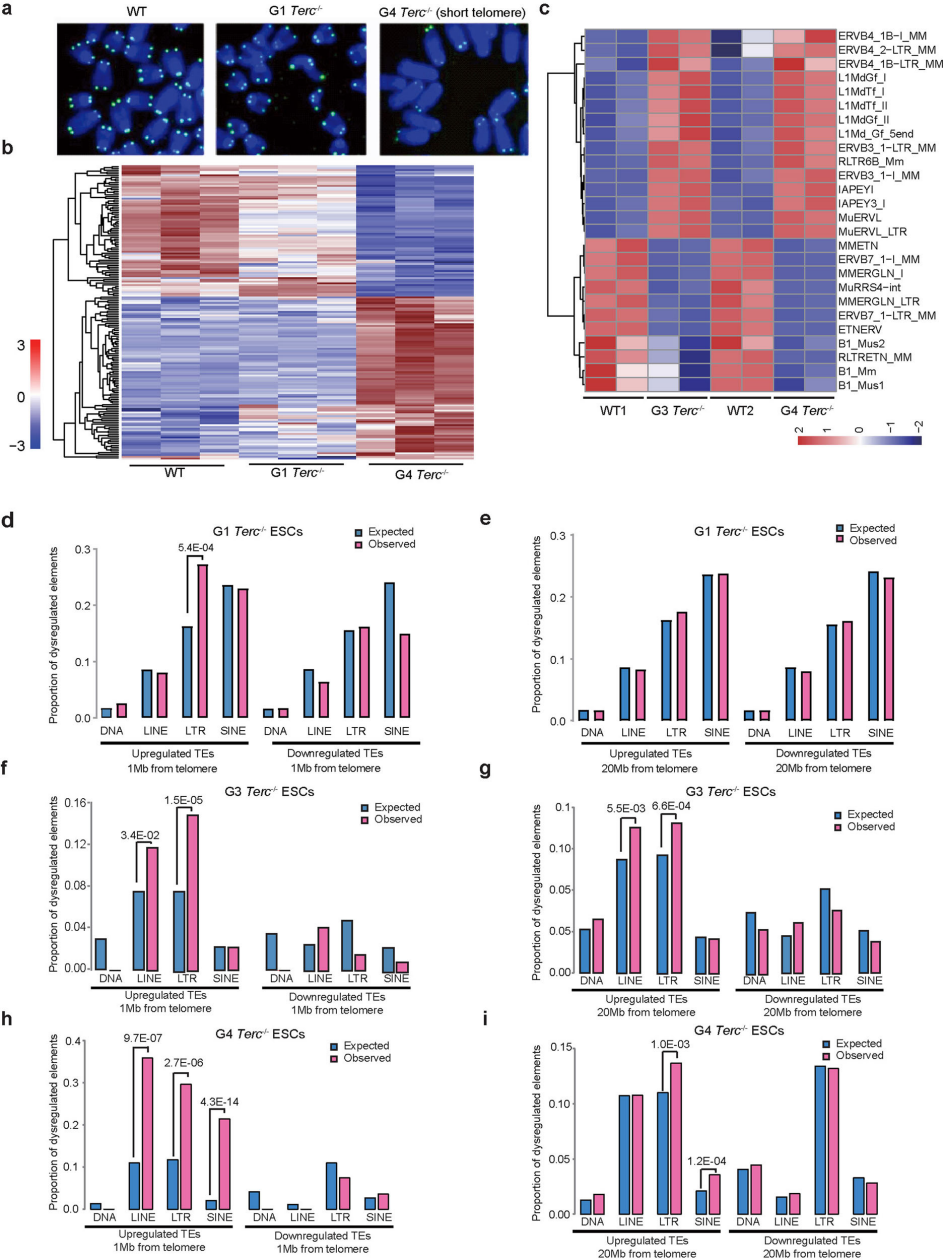
Telomere length influences the expression of subtelomeric TEs in ESCs. **a** Representative telomere Q-FISH images of WT, G1 *Terc*^−/−^ and G4 *Terc*^−/−^ ESCs. Blue, chromosomes stained by DAPI; green dots, telomeres. **b** Expression of individual TE loci in G1 and G4 *Terc*^−/−^ ESCs, compared to WT ESCs. The color bar represents the normalized *Z*-score value of the TEs. The *P* value cutoff for expression loci was < 0.05, and the fold change cutoff was > 2. Each row represents a transposon locus. **c** Common retrotransposon classes dysregulated in both G3 and G4 *Terc*^−/−^ ESCs, compared to WT ESCs. The color bar represents the normalized *Z*-score value of the TEs. Each row represents a class of TEs. **d**, **e** Enrichment of TEs in G1 *Terc*^−/−^ ESCs located within 1 Mb (**d**) or 20 Mb (**e**) from the telomere. **f**, **g** Enrichment of TEs in G3 *Terc*^−/−^ ESCs located within 1 Mb (**f**) or 20 Mb (**g**) from the telomere. **h**, **i** Enrichment of TEs in G4 *Terc*^−/−^ ESCs located within 1 Mb (**h**) or 20 Mb (**i**) from the telomere. The expected value was calculated by using the number of upregulated TEs expected to be located within the subtelomeric region by chance; the actual genomic density or distributions of each family of retrotransposons were used as the baseline. As the number of the dysregulated TEs was distinct between different generations of ESCs, the expected values are not the same in each case. The calculation method is described in detail in Materials and methods. Fisher’s exact test was used to measure the significance of enrichment.

Given that G4 *Terc*^−/−^ ESCs manifested the most retrotransposon expression changes, we mainly focused on the dysregulation of retrotransposons by short telomeres. To understand whether the activation of retrotransposons resulted from the loss of telomerase *Terc* or from the critically short telomeres themselves, we examined the expression profile of G1 *Terc*^−/−^ ESCs compared with WT ESCs at relatively early passages, in which *Terc* was deleted and telomeres were also shortened but still maintained sufficient length (Fig. 2a and Supplementary Fig. S1a, b). The TE expression profile of G1 *Terc*^−/−^ ESCs resembled that of WT ESCs, but the TE profile of G4 *Terc*^−/−^ ESCs with the shortest telomeres differed from that of G1 *Terc*^−/−^ ESCs as well as that of WT ESCs (Fig. 2b). Similar to the case in WT ESCs, retrotransposons were effectively silenced in G1 *Terc*^−/−^ ESCs, which had slightly shorter telomeres than WT ESCs (Fig. 2b), in contrast to retrotransposon activation in G4 *Terc*^−/−^ ESCs (Fig. 2b, c). Activation of retrotransposons was also found in G3 *Terc*^*−/−*^ ESCs with comparatively shorter telomeres (Fig. 2c). Retrotransposons belonging to the ERVK retrovirus family (ERVB3, ERVB4, IAPEY, and IAPEY3_I) were upregulated in both G3 and G4 *Terc*^−/−^ ESCs (Fig. 2c). Similar to ERVs, LINE1s (L1MdGf and L1MdTf) were upregulated in ESCs with shorter telomeres, compared with WT ESCs (Fig. 2c).

Next, we investigated the role of telomere length in regulating TEs by examining the genomic localization of dysregulated retrotransposons correlated with short telomeres in *Terc*^−/−^ ESCs with increasing generations from G1 and G3 to G4 (Fig. 2d–i). In contrast to the nonenrichment of downregulated TEs, upregulated long interspersed nuclear elements (LINEs) and LTRs were both enriched in the 1 Mb subtelomeric region in G3 and G4 *Terc*^−/−^ ESCs (Fig. 2f–i), suggesting that telomere length may regulate retrotransposons (Fig. 2f–i). Upregulated LINEs and LTR retrotransposons remained significantly enriched when the subtelomeric region was extended to 20 Mb in G3 *Terc*^−/−^ ESCs but not in G1 *Terc*^−/−^ ESCs (Fisher’s exact test, adjusted *P* < 0.05; Fig. 2d–g). However, when we further extended the regions to 50 Mb from telomeres, retrotransposons were no longer enriched (Supplementary Fig. S2b). These results indicate that retrotransposon activation is linked to telomere position and affected by telomere length. The downregulation of TEs may not be a direct consequence of telomere shortening. Hence, their expression was not enriched at subtelomeric regions (Fig. 2d–i). In addition, recent reports suggest that telomeres can affect distant as well as nearby chromatins^35,36^. This may explain the change in expression of retrotransposons distant from telomeres. Telomeres regulate a subset of retrotransposons instead of all retrotransposons, consistent with the concentration of upregulated elements in subtelomeric regions. Together, these results demonstrate that critically short telomeres alter the transcription of retrotransposons, regardless of cell types.

### Short telomeres increase genomic instability

**Fig. 3:**
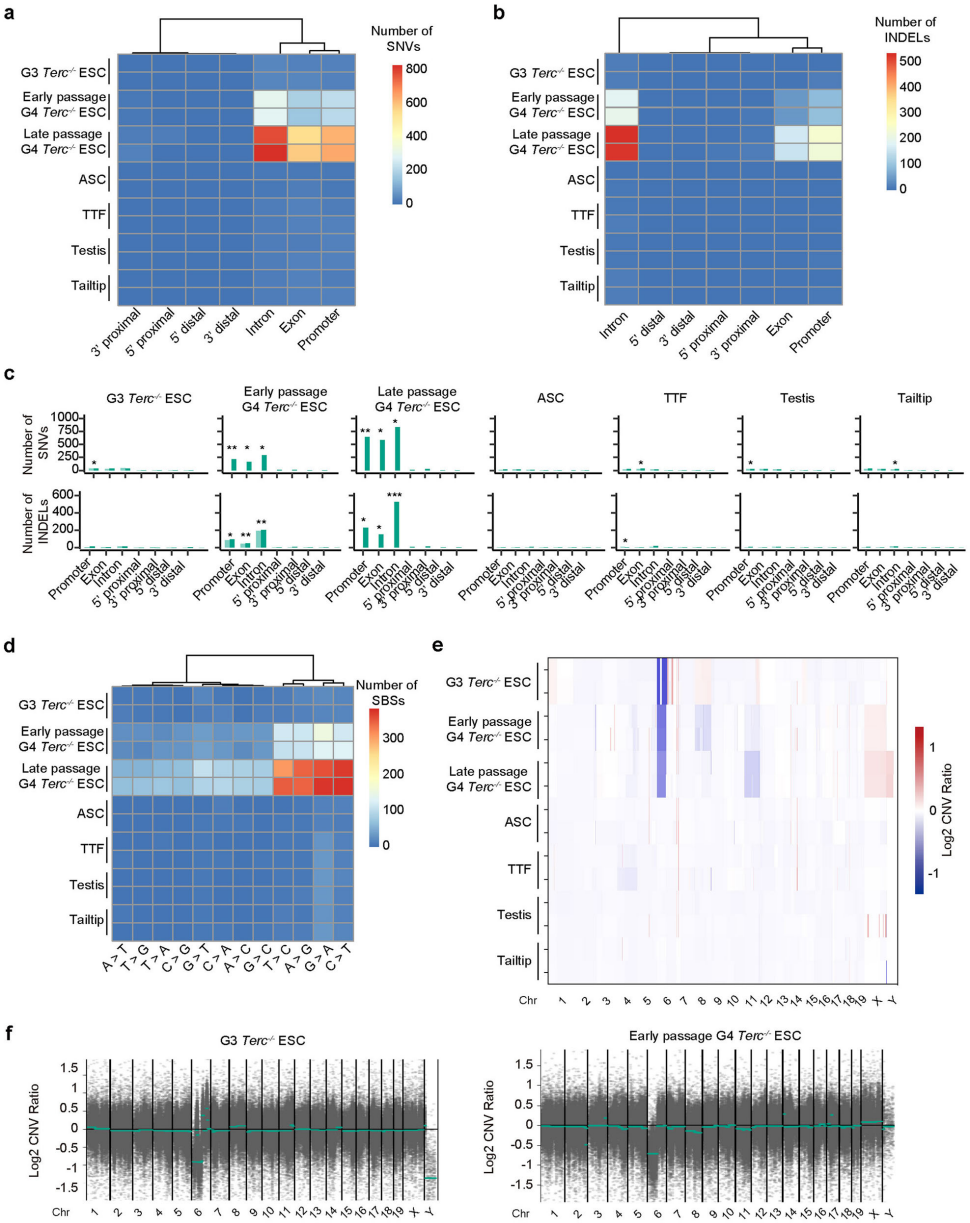
Genomic changes in various cell types or tissues with short telomeres. **a**, **b** Heatmap showing the number of SNVs (**a**) and indels (**b**) occurring in the genomes of two biological replicates according to exome-seq data. Promoter, 2 kb around transcriptional start site (TSS); 5′ proximal, 2–10 kb upstream of gene; 5′ distal, 10–100 kb upstream of gene; 3′ proximal, 0–10 kb downstream of gene; 3′ distal, 10–100 kb downstream of gene. The data were normalized to that of corresponding WT cells. **c** Bar plot of the number of SNVs and indels in the genome. Promoter, 2 kb around TSS; 5′ proximal, 2–10 kb upstream of gene; 5′ distal, 10–100 kb upstream of gene; 3′ proximal, 0–10 kb downstream of gene; 3′ distal, 10–100 kb downstream of gene. The data were normalized to that of WT cells. Green bars represent *Terc*^−/−^ cells or tissues from G3 *Terc*^−/−^ mice. *Terc*^−/−^ ASCs, which had the lowest number of total SNVs and indels, were used as the control to determine whether SNVs and indels in other types of cells or tissues were significantly increased at different genomic locations. Statistical analysis was determined by Student’s *t*-test, *P < 0.05, **P < 0.01, ***P < 0.001. **d** Heatmap showing the number of single-base substitutions (SBSs). Red indicates a larger quantity; blue indicates a smaller quantity. **e** CNV profiles of various cell types or tissues from G3 *Terc*^−/−^ mice compared to WT controls. Blue represents copy number deletion, and red represents copy number amplification. **f** CNVs in different chromosomes of G3 *Terc*^−/−^ ESCs or early-passage G4 *Terc*^−/−^ ESCs. The CNV ratio indicates the fold change in copy number relative to that in the WT sample. CNV regions were filtered with the parameter ‘depth > 10’ to remove the low-confidence regions.

Next, we asked whether the genomic integrity or stability was altered by telomere attrition. We carried out exome-seq of WT and *Terc*^−/−^ cells of various types in biological replicates. To minimize the impact of background single nucleotide variants (SNVs), we normalized the exome-seq data of *Terc*^−/−^ cells to that of corresponding WT cells. The number of SNVs was generally low in the WT tissues or somatic cells examined and did not change substantially in *Terc*^−/−^ cells with relatively short telomeres (Fig. 3a). Surprisingly, the number of SNVs markedly increased in G4 *Terc*^−/−^ ESCs at both early (P18) and late passages (P42), but only slightly increased in G3 *Terc*^−/−^ ESCs (P22), TTFs, testis and tail tip samples (Fig. 3a–c). Similarly, the number of indels did not increase in *Terc*^−/−^ somatic cells except for TTFs; rather, indels mainly increased in G4 *Terc*^−/−^ ESCs at both early and late passages (Fig. 3b). G4 *Terc*^−/−^ ESCs at late passages exhibited more SNVs and indels than those at early passages (Fig. 3a–c). Both the increased SNVs and indels were essentially associated with intron, exon and promoter regions (Fig. 3c), indicating a possible association of genomic instability with gene expression. However, genes with SNVs or indels did not exhibit expression changes at the transcription level (Supplementary Fig. S3a). We further examined the signature of SNVs. Unexpectedly, SNVs did not occur proportionately among different nucleotides. The majority of emerged SNVs are A/G or C/T SNVs (Fig. 3d), implying that the chemical structure of nucleotides may affect SNV occurrence. Increases in copy number variations (CNVs) were found in G4 *Terc*^−/−^ ESCs at both early and late passages, but were minimal in other cell types (Fig. 3e). Interestingly, a portion of chromosome 6 was deleted in both G3 and G4 *Terc*^−/−^ ESCs (Fig. 3e, f). These data suggest that mutations and genome instability are coincident with retrotransposon activation in both G3 and G4 *Terc*^−/−^ ESCs.

Additionally, we analyzed the potential effects (if any) of short telomeres on retrotransposons and genomic stability in human cancer cells. We took advantage of published data that included simultaneous measurements of telomere length and transcriptome analysis from the same single cell^5^ and analyzed selected cells with the longest and shortest telomeres. Different groups of retrotransposons were activated by shorter telomeres in human colorectal tumor cells than in tumor cells with long telomeres (Supplementary Fig. S3b, c). By using the transcriptome data^5^, we inferred that CNVs were enriched in chromosomes 1, 6, 11, or 17 of human cancer cells with short telomeres in comparison with those cells with long telomeres (Supplementary Fig. S3d). These data suggest that human colorectal cancer cells are more sensitive to telomere attrition-related retrotransposon activation and genomic instability.

### Telomere attrition alters transcription and impacts genome stability

Given that G4 *Terc*^−/−^ ESCs were derived from blastocysts developed from intercrosses of G3 *Terc*^−/−^ mice and exhibited the shortest telomeres as well as the most mutations, we focused on these cells in our analyses of genomic stability. We performed an integrated functional analysis and showed that activated genes in G4 *Terc*^−/−^ cells were enriched with similar functional terms (Supplementary Fig. S4a). The enriched pathways in G4 *Terc*^−/−^ cells were related to cell death and negative regulation of cell proliferation (Supplementary Fig. S4a). Genes related to pathways in cancer and the inflammatory response were also upregulated in G4 *Terc*^−/−^ cells but not in G3 *Terc*^−/−^ cells (Supplementary Fig. S4a–d). Interestingly, the testis, which contains spermatogonia stem cells, and ESCs exhibited enrichment of similarly upregulated pathways resulting from short telomeres. Genes that were downregulated in G4 *Terc*^*−/−*^ cells, but not in G3 *Terc*^*−/−*^ cells, were enriched in various pathways, including cellular response to DNA damage stimulus and DNA repair (Supplementary Fig. S4b, e), suggesting impairment of these biological processes by critically short telomeres in G4 *Terc*^*−/−*^ cells. Moreover, 2-cell genes were found to be enriched among the genes upregulated, but not among the genes downregulated, in telomere-shortened ESCs (Supplementary Fig. S4f). Genes related to the piRNA pathway, which is known to repress retrotransposons, were also repressed in *Terc*^*−/−*^ testis cells and late-passage G4 *Terc*^*−/−*^ ESCs (Supplementary Fig. S4f, g). This may explain the change in retrotransposon expression in *Terc*^−/−^ testes (Fig. 1c, d and Supplementary Fig. S4f, g). These data indicate that retrotransposon activation is accompanied by compromised DNA damage repair pathways and activated cancer-related pathways following telomere attrition.

### Telomere shortening reduces cell proliferation and increases apoptotic cell death

Furthermore, we evaluated the potential impact of increased DNA mutation on cell proliferation in G4 *Terc*^−/−^ ESCs. Cell proliferation slowed after the loss of *Terc* in G1, G3 and G4 ESCs (Supplementary Fig. S5a). G4 *Terc*^−/−^ ESCs showed even slower proliferation than G1 and G3 *Terc*^−/−^ ESCs (Supplementary Fig. S5a). TUNEL assays and Annexin V immunostaining revealed an increase in apoptotic cells in the G4 *Terc*^−/−^ population (Supplementary Fig. S5b–e). G1 and G3 *Terc*^−/−^ ESCs had similar rates of apoptosis, lower than that of G4 *Terc*^−/−^ ESCs (Supplementary Fig. S5b–e). Consistently, only G4 *Terc*^−/−^ ESCs, but not G1 or G3 *Terc*^−/−^ ESCs, exhibited elevated DNA damage, as evidenced by γH2AX immunofluorescence staining patterns (Supplementary Fig. S5f, g). The number of γH2AX foci was higher in G4 *Terc*^−/−^ ESCs than in G1 and G3 *Terc*^−/−^ ESCs (Supplementary Fig. S5g). These data show that telomere shortening leads to increased DNA damage and apoptosis. Given that DNA damage repair is required to maintain genome stability^37^, the increased incidences of DNA damage (Supplementary Fig. S5f, g) and suppression of the DNA repair pathway (Supplementary Fig. S4e) also explain why mutations occurred in G4 *Terc*^−/−^ ESCs.

To explore the role of LINE1s in the proliferation and telomere length maintenance, we treated G4 *Terc*^−/−^ ESCs with the reverse transcriptase inhibitor 3′-azido-3′-deoxythymidine (AZT). Indeed, inhibition of LINE1 reverse transcriptase caused cell apoptosis in both WT and G4 *Terc*^−/−^ ESCs (Supplementary Fig. S5h, i), implying that LINE1 elements do not contribute to the selective growth difference.

### Retrotransposition links critically short telomeres to genome instability

**Fig. 4:**
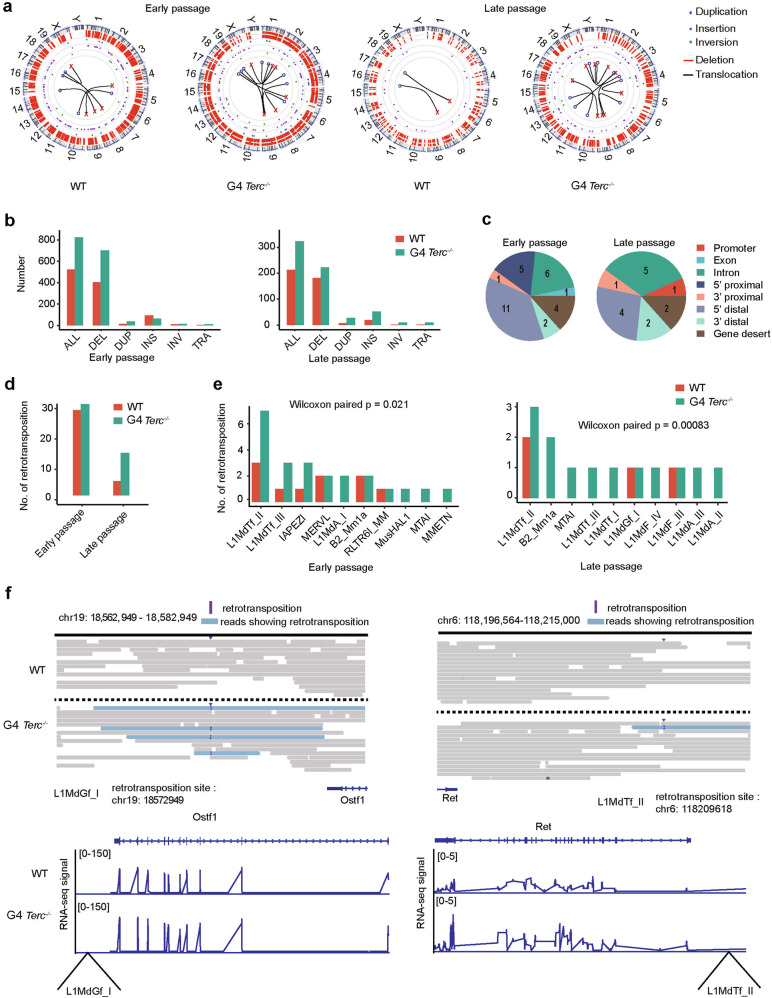
Retrotransposition driven by short telomeres. **a** Circos plot of chromatin structure variations in WT or G4 *Terc*^*−/−*^ ESCs. The chromosome number is indicated on the outside of the Circos plot. **b** Numbers of five different chromatin structure variations in WT cells or G4 *Terc*^*−/−*^ ESCs. DEL deletion, DUP duplication, INS insertion, INV inversion, TRA translocation. **c** Pie chart showing the proportion of retrotransposition events occurring in the genome. Promoter, 2 kb around TSS; 5′ proximal, 2–10 kb upstream of gene; 5′ distal, 10–100 kb upstream of gene; 3′ proximal, 0–10 kb downstream of gene; 3′ distal, 10–100 kb downstream of gene; gene desert, > 100 kb away from the nearest gene in WT cells and *Terc*^*−/−*^ ESCs at early or late passage. The number of the retrotransposition events was included on the pie chart. **d** Numbers of TE insertions in WT or G4 *Terc*^−/−^ ESCs. **e** Number of TE insertions within specific TE classes in WT or G4 *Terc*^−/−^ ESCs. **f** Examples of the locations of L1MdGf_I and L1MdTf_II insertion sequencing tags in WT and G4 *Terc*^−/−^ ESCs at late passage. The reads supporting the insertion are marked in blue, and the insertion sites are marked with a capital “I”. RNA-seq signals of *Ostf1* around the L1MdGf_I insertion site and *Ret* around the L1MdTf_II in WT and late-passage *Terc*^−/−^ ESCs are shown below the respective genomic regions.

We further performed third-generation sequencing of the mouse genome to identify whether genome stability was affected by telomere attrition. In previous work, third-generation sequencing effectively detected structural variations in the genome but exhibited only limited accuracy in the identification of CNVs and SNVs^38^. Hence, our analysis focused on the structural variations in the genome after telomere shortening. Incidences of DNA fragment deletion, duplication, insertion, inversion, and chromosomal translocation all increased in both early (P17)- and late (P42)-passage G4 *Terc*^−/−^ ESCs in comparison with respective WT ESCs (P17 or P42) (Fig. 4a). Deletions appeared to be the most frequent genomic instability events that were associated with short telomeres, whereas translocation was the least frequent event in both early- and late-passage G4 *Terc*^−/−^ ESCs (Fig. 4b). Deletion, duplication, insertion and inversion events increased across different genomic regions except for exons (Supplementary Fig. S6a), presumably because exon disruption is detrimental to gene and cell functions. Interestingly, there was a decrease in the number of chromatin structure variations in late-passage ESCs compared with early-passage ESCs (Fig. 4a, b). This could be because ESCs without mutations proliferate faster than those with more genomic defects during passaging, such that ESCs with mutations are eventually outcompeted by WT ESCs without mutations.

Furthermore, since TEs were aberrantly activated after telomere attrition, we examined whether any retrotransposition events took place. Most retrotransposons were inserted into non-exon regions such as 5′ distal and intron regions (Fig. 4c). The frequency of retrotransposition was slightly higher in G4 *Terc*^−/−^ cells than in WT cells at early passages, and the difference notably increased in G4 *Terc*^−/−^ cells at late passages. We speculate that sufficient replication time with increasing passages is required for retrotransposition to take place. The number of retrotransposition events in G4 *Terc*^−/−^ ESCs at late passages was increased compared with that of WT ESCs at the same passage, but lower than that of early-passage G4 *Terc*^−/−^ ESCs (Fig. 4d). This could be due to survival selection during the passages of ESCs. ESCs with severe genome instability (Fig. 4b–e), may undergo apoptotic cell death, as observed in G4 *Terc*^−/−^ ESCs (Supplementary Fig. S5a–e), while only cells with more stable genomes survived passaging. This was supported by the fact that telomere shortening can activate an ATM-driven DNA damage response (DDR), which may eventually lead to aneuploidy and mitotic catastrophe^39^. It is possible that the retrotransposition events observed in cells may change after passaging. Therefore, we did not consistently observe retrotransposition events occurring at the same genomic loci. Although the absolute number of retrotransposition events was lower in late-passage than early-passage G4 *Terc*^−/−^ ESCs (Fig. 4d), we noticed that retrotransposition events were much more significant in late-passage than early-passage G4 *Terc*^−/−^ ESCs compared to the corresponding WT cells (*P* = 0.00083 vs *P* = 0.021) (Fig. 4e). More active retrotransposition was associated with more SNVs and indels. Retrotransposition was prominent for young LINE1 family members (L1MdTf, L1MdA, L1MdG) (Fig. 4e and Supplementary Fig. S6b, c), but less frequent for other retrotransposons in telomere-shortened cells (Fig. 4e). Activation of LINE1 is a prerequisite for LINE1 retrotransposition. Additionally, the insertion of retrotransposons into non-gene body regions may affect gene expression. This was exemplified by L1MdGf insertion near *Ostf1* and L1MdTf insertion near *Ret* (Fig. 4f). The retrotransposition event was accompanied by the upregulation of the nearby oncogenes *Ret* and *Ostf1* (Fig. 4f), probably caused by the enhancer function of LINE1. These data suggest that increased expression of retrotransposons influences genome stability and gene expression by retrotransposition.

### Genomic 3D structure is altered in cells with critically short telomeres

**Fig. 5:**
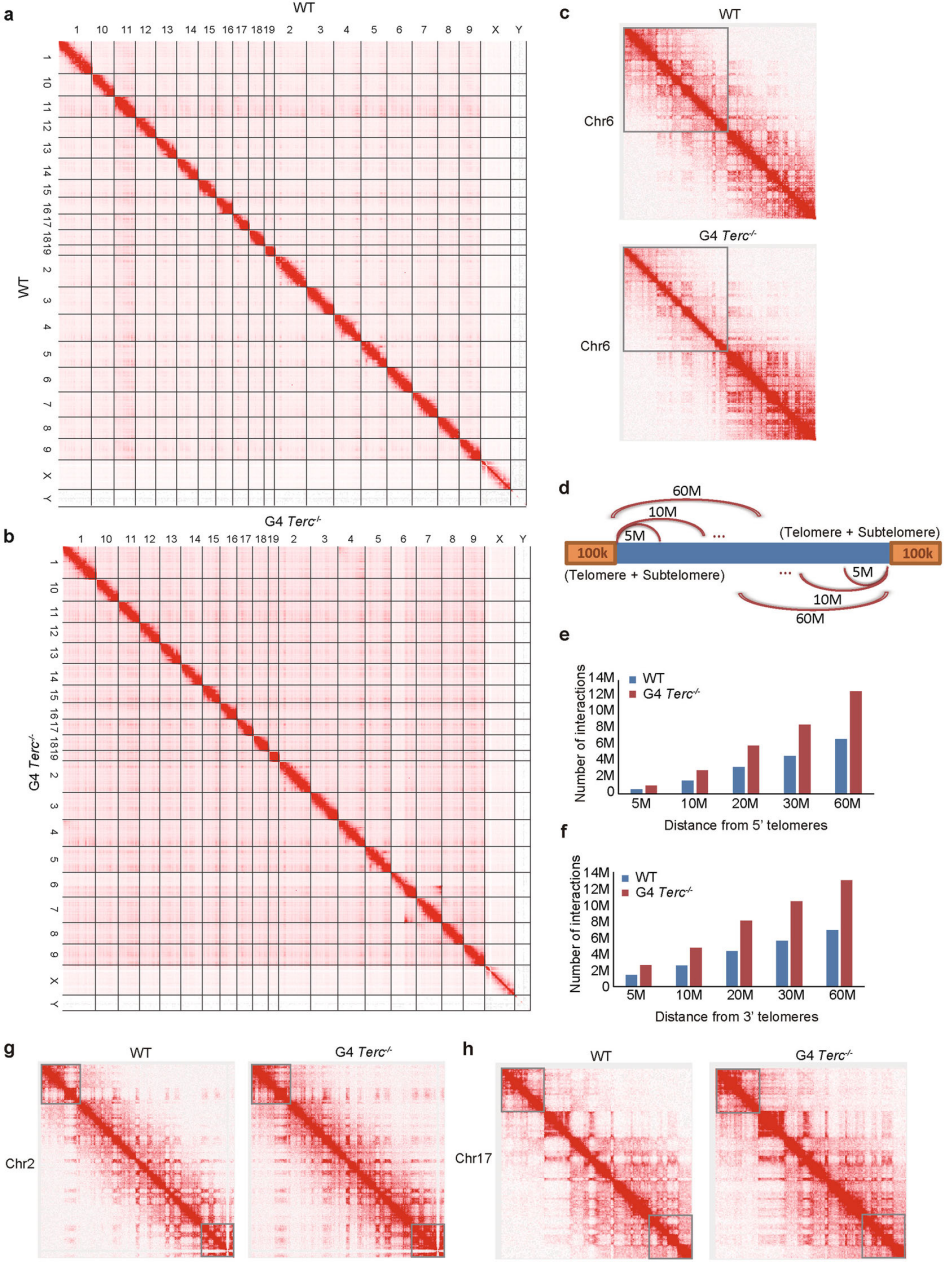
3D genomic structure changes associated with critically short telomeres. **a**, **b** Hi-C contact maps across all the chromosomes in WT (**a**) and G4 *Terc*^−/−^ ESCs (**b**). The chromosome number is indicated at the top and on the left of the figure. **c** Hi-C contact maps at 500-kb resolution across the entire chromosome 6. Gray square highlights the region truncated in chromosome 6. **d** Schematic diagram of Hi-C data to analyze the interactions of subtelomeric regions with other chromatin regions. The number of interactions was calculated according to the distance from telomere. The unit of distance is bp. **e** Number of interactions at left subtelomeric regions in WT and G4 *Terc*^−/−^ ESCs. The unit of distance is bp. **f** Number of interactions at right subtelomeric regions in WT and G4 *Terc*^−/−^ ESCs. The unit of distance is bp. **g** Hi-C contact maps at 500-kb resolution across the entire chromosome 2 in WT and G4 *Terc*^−/−^ ESCs. The grey square highlights the subtelomeric region in chromosome 2. **h** Hi-C contact maps at 500-kb resolution across the entire chromosome 17 in WT and G4 *Terc*^−/−^ ESCs. The grey square highlights the subtelomeric region in chromosome 17. The grey square highlights the subtelomeric regions with changes in chromatin interactions.

Retrotransposons have also been shown to act as enhancers that can interact with and promote the transcription of other genomic loci through chromatin looping^40,41^. It remains unclear whether telomere length influences chromatin 3D structure. Therefore, we analyzed the 3D chromatin structures of G4 *Terc*^−/−^ compared with WT ESCs by Hi-C. The deletion of chromosome 6 was again observed, as shown by the reduction in chromosome interactions for a region of chromosome 6 in comparison with other chromosomes (Fig. 5a–c), supporting the CNV change observed in the analysis of exome-seq data (Fig. 3e, f). We calculated the number of interactions at 60 Mb subtelomeric regions (Fig. 5d). Global chromatin interactions appeared to be increased by telomere shortening (Fig. 5a, b). Interestingly, the number of chromatin looping interactions obviously increased at both the 5′ and 3′ subtelomeric regions (Fig. 5e, f). This was exemplified by the cases of chromosomes 2 and 17 telomeres of G4 *Terc*^−/−^ ESCs (Fig. 5g, h). The number of subtelomeric chromosomal interactions on chromosomes 2 and 17 increased (Fig. 5g, h). To test whether the increased contacts are specific to subtelomeres, we compared the interactions of chromosome centers with those of subtelomeres. By normalizing the percentage of interactions against the total number of interactions, we found that the interactions at chromosome centers decreased after telomere shortening (Supplementary Fig. S6d, e), whereas the percentage of interactions specifically increased at subtelomeric regions (Supplementary Fig. S6d, e). Previous studies suggested a positive correlation between the number of chromosomal interactions and gene activation^42,43^. These results indicate that telomere attrition promotes chromatin interactions at subtelomeric regions, consistent with the finding that retrotransposons were upregulated at subtelomeric regions because retrotransposons can act as enhancers mediating chromosomal interactions^40,41^.

### Chromatin accessibility regulates telomere-mediated retrotransposon silencing

**Fig. 6:**
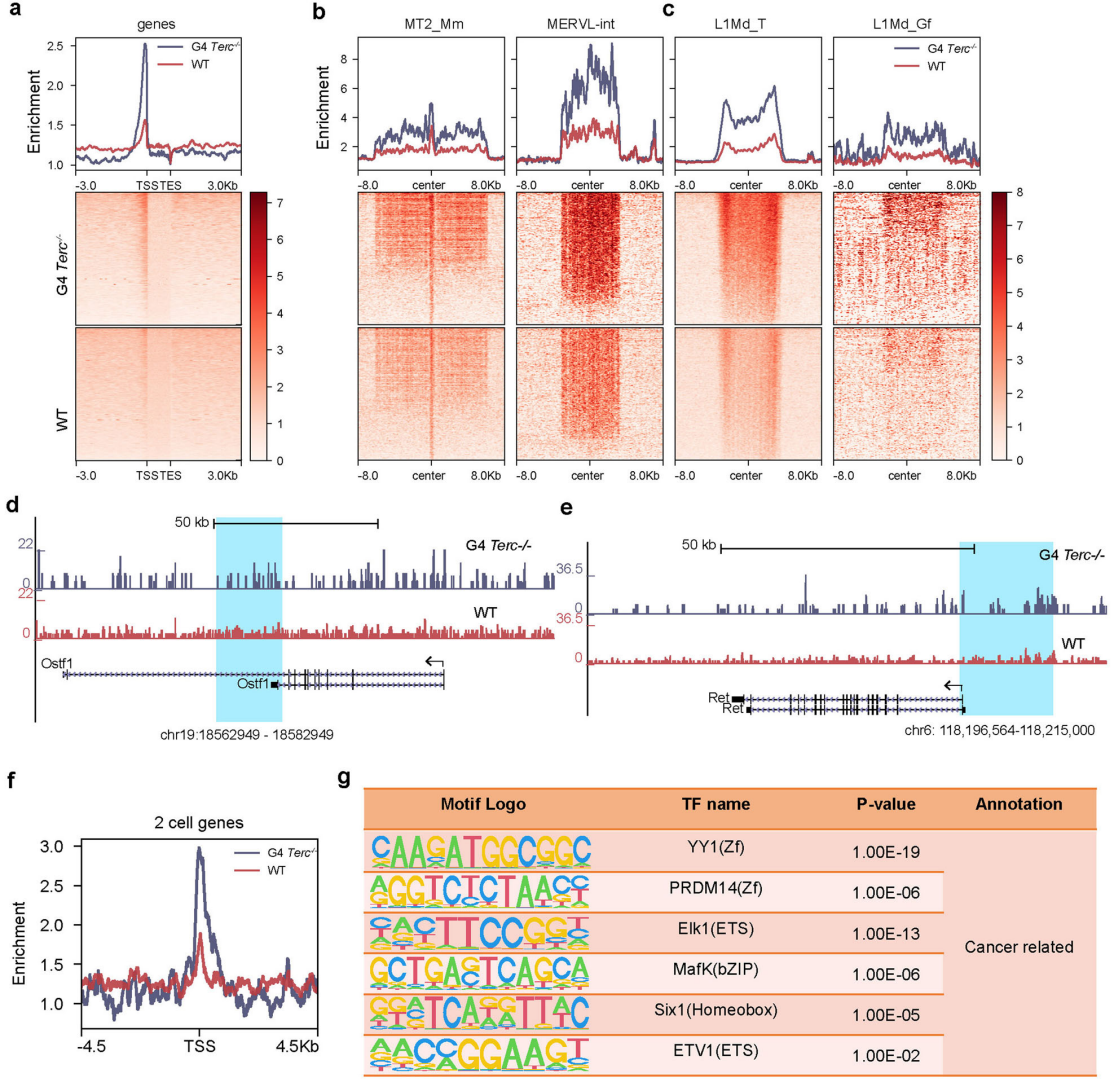
Chromatin accessibility changes in cells with critically short telomeres. **a** ATAC-seq signals of WT and G4 *Terc*^−/−^ ESC genes around the TSS. **b** ATAC-seq signals around the center of MT2_Mm and MERVL-int in WT and G4 *Terc*^−/−^ ESCs. The signal was calculated as RPKM. **c** ATAC-seq signals around the center of L1Md_T and L1Md_Gf in WT and G4 *Terc*^−/−^ ESCs. The signal was calculated as RPKM. **d** ATAC-seq signals of the *Ostf1* gene in WT and G4 *Terc*^*−/−*^ ESCs. The blue region corresponds to the LINE1 insertion. The *y*-axis represents relative RPKM of sequencing reads. **e** ATAC-seq signals of the *Ret* gene in WT and G4 *Terc*^*−/−*^ ESCs. The blue region corresponds to the LINE1 insertion. **f** Enrichment of ATAC-seq signal within 2-cell genes in WT and G4 *Terc*^*−/−*^ ESCs. **g** Enrichment of transcription factor motifs within specific ATAC-seq peaks showing increased signal in G4 *Terc*^*−/−*^ ESCs.

The expression of genes and TEs is changed by telomere attrition. We asked whether chromatin accessibility was altered by short telomeres. We performed ATAC-seq to investigate how chromatin structure regulates retrotransposons after telomere attrition. Gene TSSs became more open in G4 *Terc*^−/−^ than in WT ESCs (Fig. 6a). Moreover, G4 *Terc*^−/−^ ESCs had more ATAC-seq peaks than WT ESCs (Supplementary Fig. S7a, b), indicating that chromatin is more relaxed in ESCs with shorter telomeres. Chromatin accessibility also increased on MT2/MERVL, L1Md_T, and L1Md_Gf (Fig. 6b, c), which were upregulated in G4 *Terc*^−/−^ ESCs. Furthermore, chromatin accessibility at *Ret* and *Ostf1* increased following LINE1 retrotranspositions in G4 *Terc*^−/−^ ESCs (Fig. 6d, e), consistent with the RNA-seq data (Fig. 4f). ESCs sporadically express 2-cell genes and ERVs such as MERVL and Zscan4 in 1%–5% of the ESC population^17,18^. In agreement with increased expression levels of 2-cell genes and MERVL, promoter regions of 2-cell genes exhibited increased chromatin accessibility resulting from short telomeres (Fig. 6f). Additionally, in support of the observed gene expression changes (Supplementary Fig. S4a–d), DNA motifs associated with cancer-related transcription factors were found on the ATAC-seq peaks that were enriched in ESCs with critically short telomeres (Fig. 6g). These data indicate that elevated chromatin accessibility associated with shortest telomeres may account for the upregulation of specific genes and retrotransposons.

### Retrotransposon activation is accompanied by a change in H3K9me3 enrichment

**Fig. 7:**
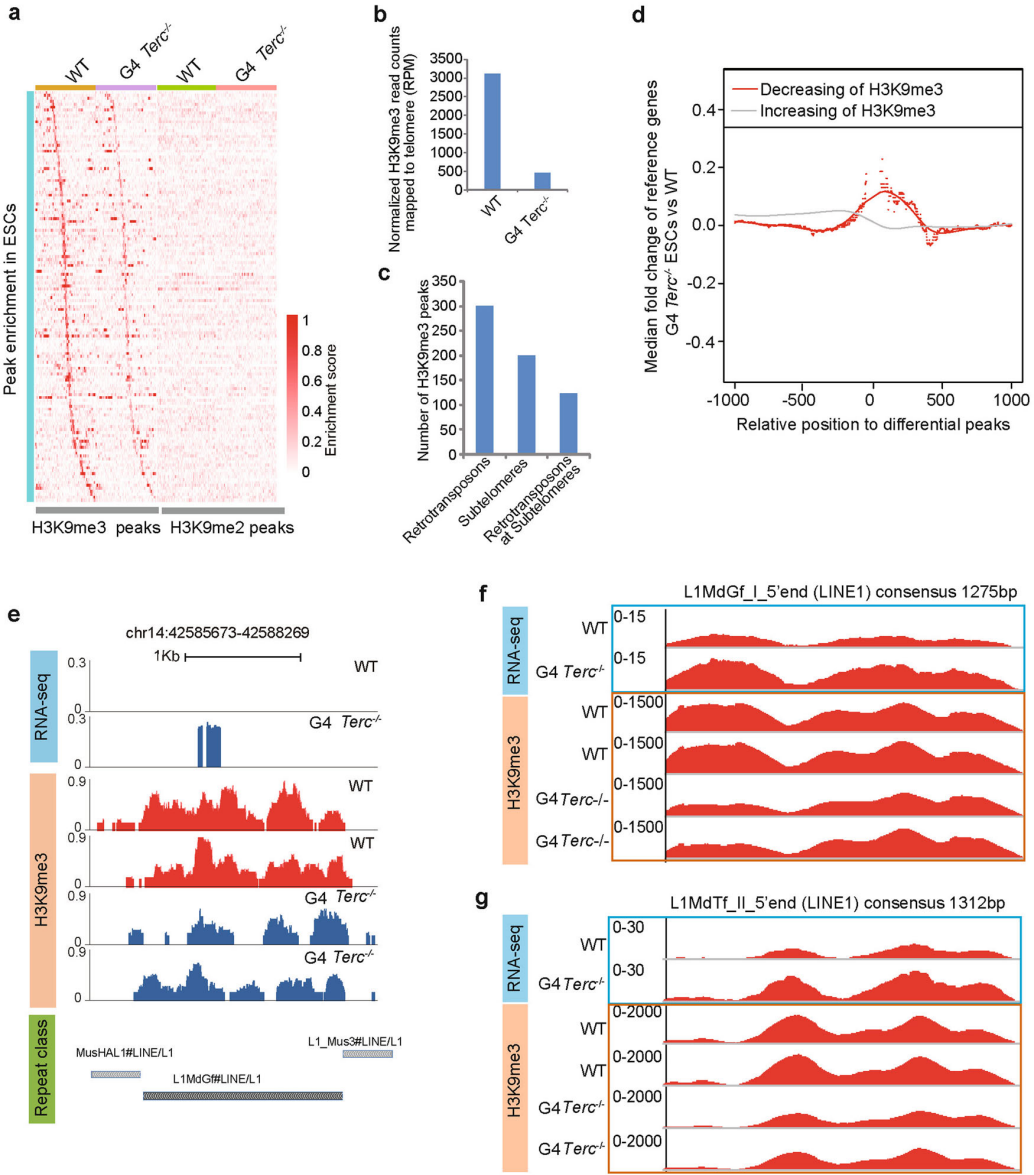
H3K9me3 regulates the retrotransposon activity induced by critically short telomeres. **a** ChIP-seq read density heatmaps for differentially enriched H3K9me3 peaks around the TSS (+/– 50 kb) and the corresponding H3K9me2 signal in WT and G4 *Terc*^−/−^ ESCs. The relative density of H3K9me3 enrichment was presented in red, as indicated by the scale bar. The heatmap was generated using NGSPLOT. **b** H3K9me3 enrichment on telomeres. The number of reads was mapped to telomere (TTAGGG) repeats and normalized to the total number of reads in each sample. The number of reads was used to indicate H3K9me3 abundance at telomeres in two biological replicates. **c** The number of downregulated H3K9me3 peaks on retrotransposons, subtelomeres and subtelomeric retrotransposons in G4 *Terc*^*−/−*^ ESCs. H3K9me3 peaks indicate their genome-wide enrichment, excluding telomere regions. **d** Metagene expression analyses of genes flanked by H3K9me3 peaks. For both the 5′ and 3′ genomic directions of H3K9me3 peaks, the median log_2_ fold changes of genes around peaks with decreasing (in red) or increasing H3K9me3 (in grey) in different window sizes are plotted. **e** L1MdGf as an example of expression and H3K9me3 change by short telomeres. **f**, **g** Coverage plot of ChIP-seq and RNA-seq reads on the consensus sequence of LINE1 family member L1MdTf (**f**) and L1MdGf (**g**). The *y*-axis indicates relative RPKM of sequencing reads. The *x*-axis indicates the full length of the corresponding retrotransposons.

To identify whether chromatin openness is associated with epigenetic changes, we performed ChIP-seq analysis of H3K9me3 and H3K9me2. We identified 512 H3K9me3 peaks that were reduced or lost in G4 *Terc*^*−/−*^ ESCs compared with WT ESCs, and none of these regions were marked by H3K9me2 (Fig. 7a). Moreover, 3160 H3K9me3 reads were mapped to telomeres in WT ESCs in contrast to only 460 H3K9me3 reads on the telomeres of G4 *Terc*^−/−^ ESCs (Fig. 7b). Among the 512 peaks with decreased H3K9me3 signals in G4 *Terc*^*−/−*^ ESCs, 200 peaks (39%) were located at subtelomeric regions, and 123 out of these 200 peaks were at retrotransposons (Fig. 7c). These ChIP-seq data further confirmed that H3K9me3 was mainly located at telomeres and subtelomeres and could mediate the silencing of a subset of genes and retrotransposons, suggesting that telomere heterochromatin may regulate the expression of retrotransposons by interacting with neighboring and distant loci.

Genes close to the peaks with decreased H3K9me3 signal tended to be upregulated in G4 *Terc*^*−/−*^ ESCs (Fig. 7d). Upregulation of the LINE1 family member L1MdGf, as revealed by RNA-seq analysis, was associated with reduced enrichment of H3K9me3 (Fig. 7e). To address whether a specific class of retrotransposons is regulated by telomere length, we mapped RNA-seq and ChIP-seq reads to the retrotransposon consensus sequences. Specific classes of retrotransposons, such as L1MdTf (LINE1) and L1MdGf (LINE1), were upregulated by telomere loss, accompanied by a reduction in H3K9me3 modification (Fig. 7f, g). The regions with decreased H3K9me3 peaks were also associated with a slightly increased ATAC-seq signal (Supplementary Fig. S7c). These results indicate the specificity of telomeres in repressing retrotransposons. Together, these findings suggest that H3K9me3 enrichment contributes at least partly to the telomeric repression of specific retrotransposons.

### Re-elongation of telomeres by the introduction of *Terc* restores telomere-mediated retrotransposable element silencing

**Fig. 8:**
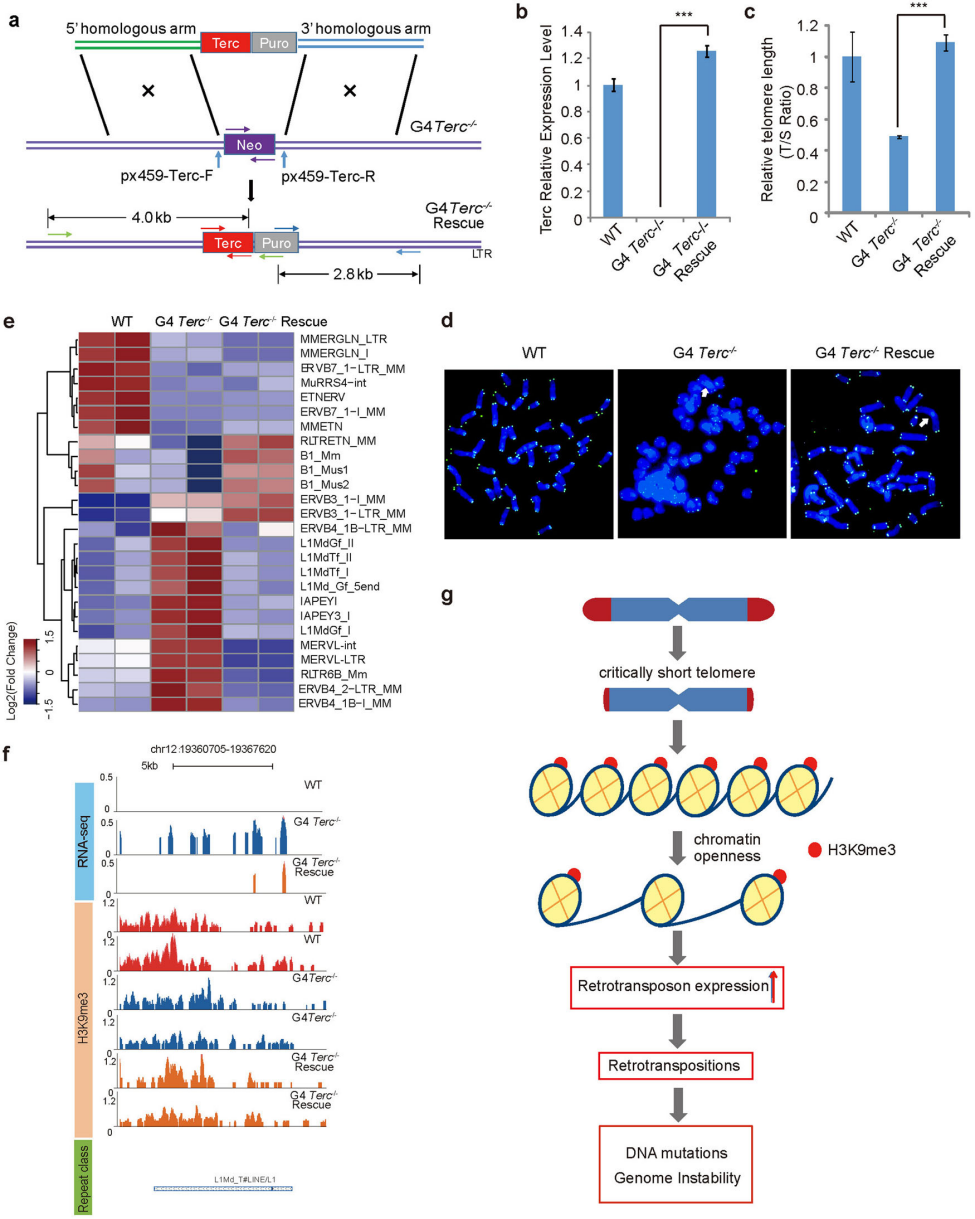
Rejuvenation of telomere length suppresses retrotransposons. **a** Illustration of the method used to knock in *Terc* into G4 *Terc*^−/−^ ESCs using CRISPR/Cas9 in combination with homologous recombination. *Terc*-puro was knocked into the *Terc*^−/−^ locus. **b** Relative expression levels of *Terc* in WT, G4 *Terc*^−/−^ and G4 *Terc*^−/−^ rescue ESCs. **c** Rescue of telomere length shown as the T/S ratio by real-time PCR. **d** Representative telomere Q-FISH images of WT, G4 *Terc*^−/−^ and G4 *Terc*^−/−^ rescue ESCs. Blue, chromosomes stained by DAPI; green dots, telomeres. White arrows indicate chromosome fusion events. **e** Expression of TEs after restoration of telomere length in G4 *Terc*^−/−^ ESCs. The bar color indicates the *Z*-score value. **f** The UCSC profile displaying the signal of the RNA-seq and H3K9me3 ChIP-seq signals around L1Md_T in WT, G4 *Terc*^*−/−*^ and G4 *Terc* rescue ESCs. The *y*-axis represents the relative RPKM of sequencing reads. Repeat classes are indicated at the bottom of the figure in green-edged boxes. **g** A simplified model of telomere-mediated silencing of retrotransposons. Critically short telomeres are correlated with a loss of heterochromatin, which subsequently leads to retrotransposon activation, promoting genome instability.

To further substantiate the role of telomeres in silencing retrotransposons, we performed rescue experiments by knock-in of *Terc* via CRISPR/Cas9 into G4 *Terc*^−/−^ ESCs (P20) to rejuvenate telomeres (Fig. 8a). We validated the knock-in of *Terc* by PCR (Supplementary Fig. S7d). A sufficient number of passages following *Terc* knock-in were necessary for elongation of telomeres and recovery of telomerase. Hence, we continued culturing the cells for 22 more passages to obtain P42 G4 *Terc*^−/−^ ESCs. Real-time PCR showed that the reduced expression of *Terc* in G4 *Terc*^−/−^ ESCs was restored in *Terc*-rescued ESCs (P42) (Fig. 8b). Telomere length was successfully rejuvenated by the introduction of *Terc* (Fig. 8c, d). A higher level of telomere signals suggested longer telomeres (green) similar to those in WT cells in contrast to the shortened telomeres and telomere signal-free ends and chromosome fusion in G4 *Terc*^−/−^ cells, indicating that the telomeres are restored in the G4 *Terc*^−/−^ rescue cells following targeted knock-in of *Terc* and recovery of telomerase (Fig. 8d). Southern blot data from TRF analysis also validated the elongation of telomeres after the introduction of *Terc* into *Terc*^−/−^ ESCs (Supplementary Fig. S1a, b). However, the chromosome fusions present in the original G4 *Terc*^−/−^ ESC line were still retained in the telomere-rejuvenated G4 *Terc*^−/−^ ESCs even though telomerase was recovered (Fig. 8d). RNA-seq analysis also demonstrated the re-suppression of the expression of retrotransposon, such as IAP members and LINE1 members, by elongated telomeres (Fig. 8e). For example, the expression of LINE1 (L1Md_T) was rescued by elongated telomeres, accompanied by a partial reduction in H3K9me3 (Fig. 8e, f). Gene expression near the H3K9me3-marked ERVB4_2-LTR was also restored after telomere elongation (Supplementary Fig. S7e). Hence, re-elongation of telomeres partially restores retrotransposon suppression.

## Discussion

We show that retrotransposons can be activated in cells with very short telomeres and that this activity is linked with genomic instability and promotion of retrotransposition-induced genomic mutations and deletion or amplification; these changes are mediated by the relaxation of chromatin structure. On the basis of our data, we propose a model whereby cells with long telomeres silence a subset of retrotransposons and maintain genome stability (Fig. 8g). After telomere attrition, chromatin accessibility is increased, and retrotransposition occurs following the activation of transposons in a subset of chromatin regions (Fig. 8g). The retrotransposition events destabilize the genome, leading to mutations through the dysregulation of DDR, inflammatory responses and cancer-related pathways (Fig. 8g).

Although telomere attrition can be found in both G3 *Terc*^−/−^ somatic cells and G1 *Terc*^−/−^ ESCs, G4 *Terc*^−/−^ ESCs exhibit the shortest telomeres as evidenced by substantial telomere signal-free ends, presumably because of the highly proliferative nature of ESCs during passaging. Nevertheless, ESCs with telomere loss rarely show chromosome fusions (Supplementary Fig. S1c), which is consistent with recent findings that telomere protection is achieved by distinct mechanisms in pluripotent cells^44,45^. When telomeres become critically short, they activate the DDR in both somatic cells and ESCs^39,46,47^. TRF2, a component of the shelterin complex, prevents telomeric DNA from being recognized as DNA damage, thus preventing the activation of DDR^48^. ESCs devoid of TRF2 at telomeres activate an attenuated telomeric DDR that is not accompanied by telomere fusions^44,45^. Although the specific mechanisms remain to be investigated, critically short telomeres also impair DNA repair pathways (Supplementary Fig. S4e). The finding that G3 *Terc*^−/−^ ESCs did not exhibit increased SNVs could be explained by the fact that DNA damage did not occur frequently in G3 *Terc*^−/−^ ESCs as in G4 *Terc*^−/−^ ESCs (Fig. 3c and Supplementary Fig. S5f, g). Furthermore, the genes downregulated in G4 *Terc*^−/−^ ESCs, but not G3 *Terc*^−/−^ ESCs, were enriched for the DNA repair pathway (Supplementary Fig. S4e). DNA repair factors can restrict LINE1 retrotransposition^49^. The undisrupted expression of DNA repair genes may be another reason that fewer mutations are observed in G3 *Terc*^−/−^ ESCs than in G4 *Terc*^−/−^ ESCs.

Moreover, short telomeres activated retrotransposons and chromosome 6 truncation occurred in both G3 and G4 *Terc*^−/−^ ESCs. However, strong DNA damage, which is indicated by high levels of γH2AX staining, occurred only in G4 *Terc*^−/−^ ESCs in which telomeres became critically short and chromosomal fusions emerged (Supplementary Figs. 1a–c and S5f, g). Given that both G3 and G4 *Terc*^−/−^ ESCs displayed chromosomal rearrangements, the increase in genomic mutations in G4 *Terc*^−/−^ ESCs is unlikely to be caused by clonal expansion of G4 cell lines. Mutations in G4 *Terc*^−/−^ ESCs were associated with increased DNA damage. Consistent with this observation, RNA-seq analysis also revealed specific downregulation of DNA damage-related genes in G4 *Terc*^−/−^ ESCs (Supplementary Fig. S4e). These results imply that the extensive DNA damage may be the reason for the emergence of DNA mutations in G4 *Terc*^−/−^ ESCs. The degree of telomere attrition affects retrotransposon activities, with critically short telomeres or telomere loss associated with increased retrotransposon activities. WT ESCs had the longest telomeres, and the shortest length was observed in ESCs after *Terc* deletion (Supplementary Fig. S1a, b). The telomeres of other tissues were also shortened but still maintained at a modest length after *Terc* deletion (Supplementary Fig. S1a, b).

Notably, the shortest telomeres were found in late-generation ESCs (G4 *Terc*^−/−^) without telomerase RNA, which provides an appropriate model for studying the impact of telomere length. Mouse cells generally have very long telomeres^50^; therefore presumably the shortest telomeres are not easily detectable in general mouse tissues or cells without telomerase. Mouse cells have much longer telomeres (50–100 kb) than human cells (5–10 kb)^50^. The telomeres in telomerase-knockout (*Terc*^−/−^) mice are shortened with increasing generations, and these mice can be bred for 6–7 generations^29^. Moreover, the length of the shortest telomeres, as evidenced by telomere signal-free ends or telomere loss, rather than the average telomere length, leads to chromosome instability^27^. In this study, telomere signal-free ends were not found in G3 *Terc*^−/−^ MEFs but were notably observed in G6 *Terc*^−/−^ MEFs, which exhibit chromosome fusion. Our study also suggested that the essential role of telomerase might not be to maintain average telomere length but rather telomere function at critically short telomeres^27^. Breeding of the knockout mice in the hybrid genetic background requires significant amount of time, extending one or two years to achieve late generations of *Terc*^−/−^ mice. Therefore, *Terc*^−/−^ mice at inbred C57BL/6 background were derived and are preferred for use. Hence, in our study, G3 *Terc*^−/−^ mice of the inbred C57BL/6 background were used for comparison with WT mice. Indeed, various somatic cells and ESCs of G3 *Terc*^−/−^ mice exhibited shorter telomeres than WT somatic cells and ESCs (Supplementary Fig. S1a–c). However, we did not observe telomere loss or critically short telomeres in somatic cell types such as TTFs readily available from G3 *Terc*^−/−^ mice (Supplementary Fig. S1a–d). We designated ESCs derived from G3 *Terc*^−/−^ blastocysts as G4 *Terc*^−/−^ ESCs which had much shorter telomeres than G3 *Terc*^−/−^ somatic cells. Moreover, the shortest telomeres as evidenced by telomere signal-free ends or telomere loss were observed in G4 *Terc*^−/−^ ESCs (Fig. 2a and Supplementary Fig. 1 c–e). Comparatively, G1 *Terc*^−/−^ ESCs also showed shorter telomeres than WT ESCs but no telomere signal-free ends (Fig. 2a), yet the retrotransposon transcription profile appeared similar to that of WT ESCs and distinct from that of G4 *Terc*^−/−^ ESCs (Fig. 2b). Similarly, low frequencies of SNVs and indels were found in both G3 *Terc*^−/−^ somatic cell types and ESCs (Fig. 3a–c). These data show that responses to telomere shortening-induced transcriptomic alteration and genomic instability can be similar in ESCs and somatic cells.

Telomeres in somatic cells or tissues in mice are generally long, and telomerase knockout shortens somatic telomeres in live mice^29^. The relatively short telomeres in somatic cells and tissues do not show evident changes in the transcription of retrotransposons and genomic stability (Figs. 1d and 3). In contrast, the shortest telomeres, despite being in ESCs, exhibit aberrant upregulation of retrotransposons, retrotranspositions and genomic instability (Figs. 1d, 3 and 4e). However, the relatively short telomeres in *Terc*^−/−^ ESCs do not induce these phenotypes (Fig. 2b, c), similar to those of *Terc*^−/−^ somatic cells. The differences in telomere shortening between somatic cells and ESCs likely arise from the rapid proliferation of ESCs, which have a doubling time of ~7–10 h, unlike somatic cells which take ~24 h to complete cell cycle. The high proliferation rate of ESCs allows them to undergo more rounds of replication within a fixed period, hence telomeres shorten more rapidly in ESCs than somatic cells without telomerase. The mutation rate was also the lowest in cells with a low rate of cell division such as spermatogonia in testes^7^. Among tested somatic tissues or cells, spermatogonia in testes rank second (behind ESCs) in terms of proliferation rate, but proliferate much faster than other somatic cell types.

G4 *Terc*^−/−^ ESCs without telomerase showed elevation of more young LINE1 elements (L1MdTf, L1MdA, L1MdG)^51^ (Figs. 2c and 4e), which can actively retrotranspose in genome, leading to genome instability, whereas other elevated TEs, such as MERVL, may not retrotranspose and initiate genome instability. However, when *Terc* was deleted, the speed of cell proliferation was reduced, and replicative senescence occurred, accompanied by telomere attrition with passaging. Sufficient telomere length is critical for cell proliferation^52^. It has also been demonstrated in somatic cells that the shortest telomere length but not the average telomere length limits cell survival^27^. Our data indicated that the shortest telomeres might also be linked to elevated retrotransposon activity, genomic instability and cellular senescence. Retrotransposon activation associated with telomere attrition also contributes to cellular senescence^23^. Hence, cell proliferation cycles affect telomere attrition and retrotransposon activation. Telomere attrition and dysfunction due to telomerase deficiency leads to aging and age-associated diseases resulting from failed protection of chromosome ends, aberrant DDR at telomeres, and genomic instability^53–56^. Short telomeres are also frequently observed during cancer development^57^. Telomere attrition can suppress tumorigenesis by promoting cellular senescence and thus causing the eventual apoptosis of these cells^58,59^. However, cancer cells, in which apoptosis pathways may be dysregulated, can survive cellular senescence despite shortened telomeres. Some cancer cells eventually lose telomere protection at chromosome ends and reach a state of genome instability, which leads to further cancer progression^60^. Our study demonstrates that in addition to the known critical role of telomeres in cancer cells, critically short telomeres may also promote cancer progression indirectly by causing aberrant upregulation of retrotransposons.

Transformed cancer cells exhibit non-telomeric reverse transcriptase activity from retrotransposons. Pharmacological inhibition or RNAi depletion of retrotransposons suppresses proliferation of cancer cells^61^. LINE1 is important for telomere maintenance in telomerase-positive cancer cells^62^. Both LINE1 and another retrotransposon, HERVK, are implicated in the in vivo tumorigenic potential of melanoma cells^61^. In addition, inhibition of LINE1 retrotransposition in mouse 2-cell embryos prevents the activation of 2-cell embryo genes^63^. We also observed the maintenance of telomeres at critically short lengths and activation of LINE1 and 2-cell embryo genes after telomerase loss (Fig. 1d and Supplementary Figs. S1a, b and S4f). Hence, it is possible that cells activate LINE1 after telomere attrition to facilitate the maintenance of telomere length. LINE1 ribonucleoprotein particles (L1-RNPs) are elevated in ALT cells^64^. L1-RNPs interact with *Terra* and L1-RNP depletion disrupts nuclear localization of *Terra*, which causes telomeric DNA damage and reduces the presence of C-circles^64^. In agreement with the findings in cancer cells, AZT-mediated LINE1 inhibition caused apoptosis in both WT and *Terc*^−/−^ ESCs (Supplementary Fig. S5h, i). Accumulation of cytoplasmic retrotransposon DNA activates the innate immunity-associated inflammatory response^22,65,66^, which may cause subsequent DNA damage. Retrotransposon activation can also cause DNA damage and vice versa^49,67,68^. It is worth investigating the mechanism by which the mutation rate is increased by critically short telomeres in the future.

Among the active retrotransposons, both ERVs and LINE1 are activated by short telomeres in ESCs (Fig. 1d, e). The activation of MERVL can be attributed to the upregulation of Zscan4 in G4 *Terc*^−/−^ ESCs^69^. Activation of ERVs and LINE1s is frequently found in cancer cells as well^70–72^. These retrotransposons can lead to the aberrant gene expression, transposition, and genomic instability. Activated ERVs may serve as enhancers/promoters to drive the expression of oncogenes in cancer cells and the expression of ERV protein products may transform cells^73^. LINE1 was reported to drive the interferon response during cell senescence and cause inflammation^22^. This may also explain the activation of genes related to the inflammatory response by short telomeres in ESCs and testes (Supplementary Fig. S4a–c). Epigenetic activation of LINE1s and their retrotransposition were also found in various human diseases, related to genome instability^74,75^. Activation of LINE1 retrotransposition may further contribute to the alteration of gene expression, genome instability, mutations^76–78^ and cancer development^72^. For example, the proto-oncogene *Ret* was activated after LINE1 retrotransposition into a nearby region (Figs. 4f and 6e). As the upregulated genes seem to be enriched in cancer-related pathways (Supplementary Fig. S4a, d), the genome of *Terc*^−/−^ cells is further destabilized after telomere attrition. This is supported by the observed deletion of a large fraction of chromosome 6 in *Terc*^−/−^ ESCs (Fig. 3e, f). The additional role for telomeres in silencing retrotransposons revealed here is fascinating. In addition to protecting chromatin ends from deterioration or fusion with other chromatin ends, telomeres maintain an inactive chromatin state, which is critical for suppressing retrotransposons to maintain genome stability.

Telomeres may also regulate retrotransposons through chromatin looping, which renders telomeres the ability to regulate genes at longer distances, e.g., 10 Mb away from telomeres^79^. Previously, Robin et al. directly mapped the interactions of specific telomere ends by using a Hi-C technique to enrich for specific genomic regions, and described how telomere length regulates gene expression long before telomeres become short enough to produce a DDR^79^. We compared the chromosomal interactions of critically short telomeres with those of long telomeres in ESCs (Fig. 5a, b). Cells with critically short telomeres exhibit a dramatic reorganization of nearby chromatin structures (Fig. 5d–g), which may create active chromatin enabling transcriptional activation of relevant retrotransposons. The increase in the number of overall chromosomal interactions suggests an active chromatin state (Fig. 5a, b). Previous findings suggest a positive correlation between the number of chromosomal interactions and gene activation^42,43^. Consistent with this notion, chromatin accessibility at the retrotransposons was elevated in ESCs with critically short telomeres (Fig. 6b, c), and heterochromatin deposition at the related foci was reduced (Fig. 7f, g). Hence, future experiments can test the hypothesis that chromatin looping may allow telomeres to control distant retrotransposons.

## Materials and methods

### Mice and tissues

The use of mice for this research was approved by the Nankai University Animal Care and Use Committee. All mice used in this study were taken care of and operated according to the relevant regulations. Mice were housed and cared for in individually ventilated cages (IVCs) on a standard 12 h light:12 h dark cycle in the sterile animal facility. Tail tips, testis, adipose and liver were dissected from G3 *Terc*^−/−^ mice and WT mice following humane euthanization.

### Cell cultures

Mouse adipose tissues were obtained from groin of G3 *Terc*^−/−^ mice and paired WT mice. They were washed with PBS, aseptically isolated and digested with 0.1% collagenase I, then placed onto culture dishes, and incubated for 4 days in ASC culture medium (knockout-DMEM containing 10% FBS, 0.1 mM non-essential amino acids, 1 mM l-glutamine, 100 U/mL penicillin and 100 µg/mL streptomycin). Mouse ASCs were digested with 0.25% trypsin-ETDA (Gibco) and transferred to new plates and maintained in ASC culture medium.

Isolation and culture of adult mouse TTFs were carried out as previously described^80^. The tails from adult G3 *Terc*^−/−^ mice and paired WT mice were peeled, minced into 1 × 1 mm^3^ pieces and cultured for 7 days in MEF medium (DMEM containing 10% FBS, 1 mM l-glutamine, and 100 U/mL penicillin and 100 µg/mL streptomycin). Cells that migrated out of the grafted pieces were mostly TTF cells. TTFs were digested with 0.25% trypsin-ETDA (Gibco) and transferred to fresh plates and maintained in MEF culture medium.

G4 *Terc*^−/−^ ESCs at the C57BL/6 genetic background were generated from blastocysts of *Terc*-deficient mice (G3 *Terc*^−/−^ mice), G3 *Terc*^*−/−*^ ESCs from G2 *Terc*^−/−^ mice and G1 *Terc*^*−/−*^ ESCs from *Terc*^+/−^ mice^32^. WT ESCs of the same C57BL/6 genetic background served as controls. ESCs were cultured as previously described^32^. The ESC culture medium consisted of knock-out DMEM (Invitrogen) containing 20% FBS (HyClone), 1000 U/mL mouse leukemia inhibitory factor (LIF; ESG1107, Millipore), 0.1 mM non-essential amino acids, 0.1 mM β-mecaptoethanol, 1 mM l-glutamine, penicillin (100 U/mL) and streptomycin (100 μg/mL). For the culture of ESCs, the medium was changed daily, and cells were routinely passaged every two days using 0.25% trypsin-EDTA (Invitrogen, 25200-072).

### Generation of *Terc* knock-in ESC lines

For *Terc* knock-in, the *Terc* repair donor sequences were based on the mouse genomic sequence and information^29^. The donor vector contained the *Terc* gene flanked by 5′ (left) and 3′ (right) homology arms. In addition, there was a puro-resistance cassette for drug selection between the *Terc* gene and the 3′ homology arm. The DNA fragments were individually amplified using the proper primers and then cloned into one vector with the proper enzymes. G4 *Terc*^−/−^ ESCs were transfected with two PX459 plasmids and *Terc* repair donor plasmids using Lipofectamine 2000 transfection reagent (Invitrogen). Twenty-four hours later, 2 µg/mL puromycin was applied for 7 days to select positive clones. Single-cell clones were picked, and then the genomic DNA was extracted. To detect whether *Terc* was properly repaired, PCR was performed with primers as described (Supplementary Table S1).

### Telomere Q-FISH

Telomere length and function (telomere integrity and chromosome stability) were estimated by Q-FISH as previously described^53,81^. Cells were incubated with 0.3 μg/mL nocodazole for 3 h to enrich cells at metaphase. Chromosome spreads were made using a routine method. Metaphase-enriched cells were exposed to hypotonic treatment with 0.075 M KCl solution, fixed with methanol: glacial acetic acid (3:1), and spread onto clean slides. The slides were washed in PBS for 15 min, and the chromosomes were fixed in 4% formaldehyde in PBS for 2 min and washed three times in PBS. The slides were then dehydrated in 70%, 90%, and 100% ethanol for 5 min each time and subsequently air-dried. Telomeres were denatured at 80 °C for 3 min and hybridized with an FITC- or Cy3-labeled (CCCTAA)_3_ peptide nucleic acid (PNA) probe at 0.5 μg/mL (Panagene, Korea) for 2.5 h at 37 °C in a humidified chamber. Chromosomes were counter-stained with 0.5 μg/mL DAPI. Fluorescence from chromosomes and telomeres was digitally imaged on a Zeiss Imager Z2 microscope with FITC/DAPI or Rhodamine/DAPI filters using AxioCam and AxioVision software 4.6. For quantitative measurements of telomere length, telomere fluorescence intensity was integrated using the TFL-TELO program (kindly provided by P. Lansdorp).

### TRF analysis by Southern blot

The average TRF length was determined according to the commercial kit (TeloTAGGG Telomere Length Assay, 12209136001; Roche Life Science). Genomic DNA was extracted by traditional phenol:chloroform:isoamyl alcohol method. 3 μg DNA was digested with *Mbo*I (NEB) overnight, and the DNA fragments were separated by 0.8% agarose gel for 16 h at 6 V/cm in 0.5× TBE buffer using CHEF DR-III pulse-field system (Bio-Rad). Gels were denatured, neutralized, and transferred to nylon membranes (RPN2020B; GE Healthcare) for 48 h. The membrane was hybridized with digoxigenin (DIG)-labeled telomere probe at 42 °C overnight and incubated with the anti-DIG-alkaline phosphatase antibody. Telomere signals were detected by chemiluminescence after the addition of the substrate solution on membrane. Telomere length was measured by TeloTool software.

### Measurement of telomere length by quantitative real-time PCR

Quantitative real-time PCR was used to measure relative telomere lengths (RTLs) as previously described^82^. Genomic DNA was extracted from cells using the DNeasy Blood & Tissue Kit (Qiagen). Average telomere length was measured from total genomic DNA using a real-time PCR assay that was modified to measure mouse telomeres. For each sample, 20 ng of genomic DNA was used in each reaction. PCR reactions were performed on an iCycler MyiQ2 Detection System (Bio-Rad) using telomeric primers and primers for the reference control gene (mouse 36B4 single-copy gene). For each PCR reaction, a standard curve was made by serial dilutions of known amounts of mouse genomic DNA. The telomere signal was normalized to the signal from the single-copy gene to generate a T/S ratio, which was indicative of relative telomere length. The primers used were listed in Supplementary Table S1.

### Treatment of ESCs with LINE1 reverse transcriptase inhibitor

Zidovudine (AZT) is a nucleoside reverse transcriptase inhibitor. The drug was obtained from Sigma (A2169) and prepared on the day of use. ESC colonies were treated with 10 μM AZT.

### Apoptosis by TUNEL assay

Apoptosis was revealed by catalytically incorporating fluorescein-12-dUTP at 3′-OH DNA ends with the terminal deoxynucleotidyl transferase recombinant enzyme (rTdT) using a commercial kit (G3250, Promega). Briefly, after being fixed in 4% paraformaldehyde, washed in PBS, and permeabilized in 0.2% Triton X-100, cells were incubated with rTdT buffer at 37 °C for 1 h, immersed in 2× SSC for 15 min to stop the reaction, stained with DAPI for 15 min, washed in PBS, mounted in Vectashield, and immediately analyzed under a fluorescence microscope using FITC/DAPI filters.

### Annexin V immunostaining

Annexin V staining was performed using the Annexin V-FITC Apoptosis Detection Kit (C1062, Beyotime). Briefly, cells were cultured on cover slips. Cell culture medium was aspirated and washed once with PBS. Annexin V-FITC binding solution was added and mixed gently. Then propidium iodide (PI) staining solution was added and mixed gently. Slides were incubated at room temperature in the dark for 20 min. Samples were loaded in Vectashield medium (Vector) to prevent photo bleaching and imaged under Zeiss Axio Imager Z2 microscope.

### Immunofluorescence microscopy of DNA damage

Immunofluorescence staining was performed as previously described^32^. Cells were washed in PBS, fixed in 3.7% paraformaldehyde, permeabilized with 0.1% Triton X-100 for 30 min at room temperature, blocked with blocking solution (3% goat serum and 0.1% BSA in PBS) for 2 h at room temperature, and incubated overnight at 4 °C with primary antibodies against γH2AX (sc517348, Santa Cruz). After three washes with blocking solution, the cells were incubated with a secondary antibody 488 goat anti-mouse IgM (A21042, Invitrogen) for 2 h followed by washing three times with PBS. Nuclei were stained with DAPI (Sigma) and samples were loaded in Vectashield medium (Vector) to prevent photo bleaching. Fluorescence images were captured using a Zeiss fluorescence microscope (LSM 800 with Airyscan) and ZEN software. Positive control for DNA damage was set as treatment of WT ESC with H_2_O_2_ for 1 h.

### Cell proliferation assay

ESCs (1 × 10^5^) were plated in 12-well plates and counted every 24 h. For count by passages, 1 × 10^5^ ESCs were plated in 12-well plates and passaged into new 12-well plates every 2 days.

### RNA-seq analysis

Total RNA extracted from WT and *Terc*^−/−^ cells and tissues was used for RNA-seq library preparation. Sequencing libraries were constructed by Novogene and sequenced following standard protocols. Paired-end mRNA-seq data consisted of read lengths of 150 bp for each sample, with two biological replicates for each line. The raw reads were first aligned to the mouse reference genome (mm10) using the STAR software^83^ with the default parameters. DEGs were calculated by the Cufflinks software^84^ and the expression levels were represented by the RPKM (Reads Per Kilobase per Million mapped reads). DEGs were defined based on the fold change in expression levels and *P* value. If the absolute fold change was > 2 and *P* value < 0.05, genes were considered to be differentially expressed. For the comparison of the function of the DEGs in the seven cell types, we used the Metascape website (https://metascape.org/gp/index.html#/main/step1).

For analysis of retrotransposons, the raw reads were first aligned to the consensus sequence of TEs which were downloaded from the REPBASE using the STAR software^83^. Only repeat elements that exist in the mouse genome were used. The number of the retrotransposons was counted based on the BAM files. Differentially expressed retrotransposons were calculated using the software DESeq2^85^. The cutoff for the differentially expressed retrotransposons was fold change > 1.5 and *P* value < 0.05. The volcano plot of the DEGs and the MA plot of the differentially expressed retrotransposons were represented by the R package ‘ggplot2’. The PCA (principal component analysis) of the retrotransposons were plotted by the R package ‘gmodels’. The heatmap of the differentially expressed genes and retrotransposons was represented by the R package ‘gplots’.

For calculating TE enrichment in the subtelomeric region, RNA-seq reads were first aligned to the mouse genome (mm10). A significant number of upregulated TE instances were identified by analyzing uniquely mapped reads of TEs between WT and *Terc*^−/−^ samples using DESeq2. A genomic instance of TEs would be excluded if they were only covered by < 5 uniquely mapped reads. For each transposon superfamily, the upregulated instances on each chromosome were classified into subtelomere adjacent and subtelomere non-adjacent instances according to a distance threshold (1 Mb and 20 Mb). The expected value is calculated using the number of up-/downregulated retrotransposons located at telomere region by chance. The observed value was calculated using the number of up-/downregulated retrotransposons at 1 Mb/20 Mb. Fisher’s exact tests were used to evaluate the significant enrichment of up-/downregulated instances associated with the telomeric region.

In the analysis of RNA-seq from colorectal cancer cells, the single-cell sequencing data were obtained from the public database under accession number SRP113436^5^. These cells were derived from the same tumor, and the analysis of telomere length and transcriptome was performed on the same single cell with previously described method^5^. We downloaded the sequencing data of 23 cells with the shortest telomeres and 50 cells with the longest telomeres. The raw data were mapped to the human consensus sequence from the Repbase database with STAR software. Only repeat elements that exist in the human genome were used. The number of the retrotransposons was counted by the self-written script based on the BAM files. Differentially expressed retrotransposons were calculated using the software DESeq2. The cutoff for the differentially expressed retrotransposons was fold change > 1.5 and *P* value < 0.05. For the colorectal cancer cells, we used InferCNV software to estimate the CNVs.

### Exome sequencing and analysis

Sample genomic DNA was extracted, and the exome-seq library construction and sequencing were all carried out by Beijing Novogene Company as follows. Paired-end DNA libraries were prepared according to the manufacturer’s instructions (Agilent) for two biological replicates. Genomic DNA from cell samples was sheared into 180–280 bp fragments by Covaris S220 sonicator. Ends of genomic DNA fragments were repaired and adenylated. Both ends of genomic DNA fragments were ligated with paired-end adaptors (Illumina) and a single ‘T’ base overhang, and purified using AMPure SPRI beads from Agincourt. The adaptor-modified genomic DNA fragments were enriched by six cycles of PCR using SureSelect Primer and SureSelect ILM Indexing PreCapture PCR Reverse Primer. The concentration and size distribution of the libraries were determined on an Agilent Bioanalyzer DNA 1000 chip. Whole-exome capture was carried out using SureSelect Mouse All Exon V1 Agilent 5190-4642. An amount of 0.5 μg prepared genomic DNA library was hybridized with a capture library for 5 min at 95 °C followed by 24 h at 65 °C. The captured DNA_RNA hybrids were recovered using DynabeadsMyOne Streptavidin T1. Capture products were eluted from the beads and desalted using QIAGEN MinElute PCR purification columns. The purified capture products were then amplified using the SureSelect ILM Indexing Post Capture Forward PCR Primer and PCR Primer Index (Agilent) for 12 cycles. After DNA quality assessment, the captured DNA library was sequenced on the Illumina Hiseq2000 sequencing platform (Illumina) according to the manufacturer’s instructions for paired-end 150 bp reads (Novogene). Libraries were loaded onto paired-end flow cells at concentrations of 14–15 pM to generate cluster densities of 800,000–900,000/mm^2^ using Illumina cBot and HiSeq paired-end cluster kit.

Analysis of genomic instability of telomere-shortened cells was performed by normalization to corresponding WT cells, according to the protocols specified for mouse cells^86^. Briefly, BWA was used to map clean reads to the mouse mm10 reference genome to obtain the Bam file. Picard and SAMtools were used to sort and process the content of the file, and to generate a final Bam file for calculating sequencing quality (sequencing depth and coverage). GATK4.1.9 was used to recalibrate the base quality score^87^. Mutect2 in GATK4.1.9, which is recommended in this protocol, was used for filtering SNVs and indels^86^. Refseq was used to further annotate the file and obtain the various locations and related information. CNVkit v0.9.7^88^ was used to detect the sequencing information of CNVs and WT cells served as control.

### Third-generation genome sequencing

High molecular weight genomic DNA of each individual was extracted from population cells using HiPure Tissue & Blood DNA Kit (D3018-03, Angen). We performed Nanopore genome sequencing, which represents a robust technology in the DNA sequencing field, producing long-read sequencing data^89^. DNA repair, end repair, and adapter ligation were conducted during library preparation, and 2 μg DNA was fragmented by g-TUBE (Covaris). DNA repair was performed using NEBNext FFPE DNA Repair Mix (M6630L, NEB). End repair was performed using NEBNext Ultra II End Repair/dA-Tailing Module (E7546L, NEB). Adapter ligation was performed using the NEBNext Blunt/TA Ligase Master Mix (M0367L, NEB) and Ligation Sequencing Kit 1D (SQK-LSK109, Oxford Nanopore Technologies). DNA was purified at the end of each step using Agencourt AMPure XP beads (A63882, Beckman Coulter). DNA was quantified via a Qubit Fluorometer 2.0 (Thermo Fisher Scientific, Waltham, MA, USA). Long-read sequencing was carried out using a PromethION sequencer and 1D flow cell with protein pore R9.4.1 1D chemistry according to the manufacturer’s instructions. Reads were base-called in batches by guppy v3.2.8 using the default parameters during sequencing.

Reads with an average 8 kb length were obtained and a total of 60 Gb data were analyzed for WT ESC and G4 *Terc*^−/−^ ESC genomes. Reads from whole-genome sequencing were mapped with NGLMR according to published method^90^. Genomic structural variants were identified by Sniffles^90^. Structural variants were calculated after normalization to that of MEF for both WT and G4 *Terc*^−/−^ ESCs.

### ATAC-seq analysis

The sequencing reads were trimmed with the Cutadapt software with the parameter “-a CTGTCTCTTATA”. Then the reads were mapped to the mouse genome mm10 with the Bowtie2 software^91^, and then the reads, which were mapped to the mitochondria were removed. The PCR duplications were also removed using the Picard tool (https://broadinstitute.github.io/picard/). The read counts were normalized with RPKM for the following analysis. The profiles of the ATAC signal were plotted with deeptools^92^. The ATAC-seq signals were visualized using the UCSC genome browser. The peaks were called by the software MACS2 (https://pypi.org/project/MACS2/) with the parameter “-f BAMPE”, and then the peaks were annotated with the R package “ChIPSeeker”^93^.

### ChIP-seq

ChIP-seq was performed as previously described^94^. Briefly, cells were first fixed with 1% paraformaldehyde, and then lysed and sonicated to achieve the majority of DNA fragments at 100–1000 bp. DNA fragments were then enriched by immunoprecipitation with H3K9me3 (ab8898, Abcam), and H3K9me2 (ab1220, Abcam), and Dynabeads M280 (Life Technologies). The immunoprecipitated material was eluted from the beads by heating for 30 min at 68 °C. Then, samples were incubated with Proteinase K at 42 °C for 2 h followed by 67 °C for 6 h to reverse crosslinking. The samples were extracted with phenol:chloroform:isoamyl alcohol, followed by chloroform, ethanol precipitation, and resuspension in TE buffer. The sequencing libraries were prepared using reagents from the Novogene Corporation. Each library was subsequently sequenced, resulting in ~20 million reads (125 bp). The sequencing results were mapped to mouse genome assembly mm10. For reads mapped to repetitive sequences, uniquely mapped reads were used.

### ChIP-seq data analysis

For ChIP-seq data analysis, the raw ChIP-seq reads were aligned to mouse genome assembly (mm10) by BOWTIE2^91^ with the parameter “--local -k 1”. This setting would ensure that if more than one equivalent best alignment was found, only one of those hits would be randomly reported. Therefore, instead of excluding reads from repetitive elements, multiple-hit reads are evenly distributed over highly similar repeat elements across the genome. The peaks of H3K9me3 were called using DFilter (v1.5)^95^ with the “ks = 20 -lpval = 3 –nonzero” setting. DESeq2 was used to identify differential peaks. The density plot of ChIP-seq reads was plotted with NGSPLOT^96^.

For the generation of H3K9me2 and H3K9me3 enrichment heatmaps, H3K9me3 and H3K9me2 peaks were mapped to the repeat consensus sequence from Repbase, and the relative enrichment of the H3K9me3 signals as the number of reads of H3K9me3 or H3K9me2 ChIP-seq divided by the number of reads of ChIP input. The DESeq2 was used to determine the log_2_ fold change of enrichment and measure the significance of enrichment. We mapped reads directly to TTAGGG repeats, the reads that match (TTAGGG)_n_ with the parameter -D 15 -R 2 -N 0 -L 20 -i S,1,1.15 using bowtie2 software were defined as telomeric.

### In situ Hi-C library preparation

Cells were fixed in 2% formaldehyde for 10 min, and the reaction was quenched by the addition of glycine (0.125 M final concentration). After centrifugation (300× *g*, 10 min), cells were washed with ice-cold PBS, precipitated, and the supernatant was removed. Cell pellets were incubated in 1 mL ice-cold lysis buffer (10 mM Tris-HCl, pH 8.0, 10 mM NaCl, 0.2% Igepal CA-630, 1× protease inhibitor (Roche) for 30 min on ice. Nuclei were collected by centrifugation at 2500× *g* for 5 min at 4 °C. Cell pellets were resuspended in 50 µL of 0.5% SDS, followed by incubation at 65 °C for 10 min. Then, 145 µL H_2_O and 25 µL of 10% Triton X-100 were added to quench the SDS and incubated at 37 °C for 15 min. To digest chromatin, 400 U of *Dpn*II (NEB) were added, and samples were incubated at 37 °C overnight with continuous agitation (900 rpm). *Dpn*II was then inactivated at 65 °C for 20 min. To fill in the biotin to the DNA, 1.5 µL 10 mM dCTP, 1.5 µL 10 mM dGTP, 1.5 µL 10 mM dTTP, 37.5 µL 0.4 mM biotin-14-dATP (Invitrogen) and 40 U Klenow (NEB) were added to the solution and the reaction was carried out at 37 °C for 1.5 h with rotation. Blunt-end ligation was performed by adding 900 µL of ligation mix (663 µL water, 120 µL of 10× NEB T4 DNA ligase buffer, 100 µL of 10% Triton X-100, 12 µL of 10 mg/mL BSA and 5 µL of 400 U/µL T4 DNA ligase), followed by overnight incubation at 16 °C. The reversal of crosslinks (65 °C, 4 h in the presence of Proteinase K), was followed by RNase A treatment and two sequential phenol chloroform extractions of DNA. DNA was sheared to 200–400 bp with Diagenode Bioruptor pico and double size selected on AMPure XP beads (Beckman Coulter). Biotin was removed from unligated restriction fragment ends by incubating DNA with T4 DNA polymerase (NEB) for 4 h at 20 °C in the presence of dATP and dGTP. The biotin-labeled DNA was then pulled down with 150 µL DynabeadsMyOne Streptavidin T1 (Life Technology) in binding buffer (5 mM Tris, pH 8.0, 0.5 mM EDTA, 1 M NaCl) for 30 min at room temperature. Sequencing library preparation was performed on beads, including end repair, dATP tailing and adaptor ligation, using NEBNext Ultra DNA Library Prep Kit for Illumina (NEB) according to the manufacturer’s instructions. 8–12 cycles of PCR amplification were performed with NEBNext Q5 Hot Start HiFi PCR Master Mix (NEB). Finally, size selection was done with AMPure XP beads and fragments ranging from 200 to 600 bp were selected. All libraries were sequenced on Illumina HiSeq X Ten.

### Hi-C data analysis

Paired-end FASTQ files were controlled for quality, mapped to the reference genome (mm10) and converted into interaction matrices using Hi-C-Pro (V2.11.1)^97^ using pipeline code available at https://github.com/nservant/HiC-Pro. We used 200 kb bin sizes to generate the raw and ICE (iterative correction and eigenvector decomposition) normalized matrix^97^. The valid pair files, were transformed to Hi-C format files which were ready for juicer (V1.9.8) for exploratory visualization.

For detection of differential chromatin interactions in subtelomeric regions, statistical analysis was performed. The 100 kb at the end of each chromosome, which is located on the heterochromatin region, was excluded for its rarely detected reads. Then we counted the number of chromatin interaction reads mapped at the end of each chromosome (5 M, 10 M, 20 M, 30 M and 60 M, respectively), including chromatin interactions both within the region and with other regions. Left and right subtelomeric regions were calculated, respectively.

### Statistical analysis

Statistical analysis was performed using Excel. Data are presented as mean ± SEM from biological triplicate data unless noted otherwise. A two-tailed paired Student’s *t*-test was used to estimate the difference between the two groups. The data is expected to be normally distributed, and the variance is expected to be similar between the groups that are being statistically compared.

## Supplementary information

Supplementary information

## Data Availability

The whole exome-seq data for *Terc*^−/−^ tissues and G3/G4 *Terc*^−/−^ ESCs have been uploaded to the SRA with accession number PRJNA764419. The long read data have been uploaded to the SRA with accession number PRJNA764424. The RNA-seq data of the seven tissues/cell types have been uploaded to the GEO under accession number GSE184444. The ATAC-seq data have been uploaded to the GEO with accession number GSE184442. H3K9me3 ChIP-seq data were deposited in the GEO under GSE100168 and H3K9me2 ChIP-seq data in the SRA under PRJNA794026, and G3/G4 *Terc*^−/−^ ESCs RNA-seq data were deposited in the GEO with accession numbers GSE118027 and GSE100168. The WT, G1, G4 *Terc*^−/−^ RNA-seq data accession number in the GEO is GSE192876. The Hi-C data accession number in the GEO is GSE210871.
