# Supplementary material for: Critically short telomeres derepress retrotransposons to promote genome instability in embryonic stem cells
Source: Cell Discov. 2023 May 2;9:45. doi: 10.1038/s41421-023-00538-y (PMC10154409; doi:10.1038/s41421-023-00538-y)
Supplement: Supplementary file 1 — Supplementary information [file 41421_2023_538_MOESM1_ESM.pdf]

Supplementary Information

Supplementary Figures S1-7

Supplementary Table S1

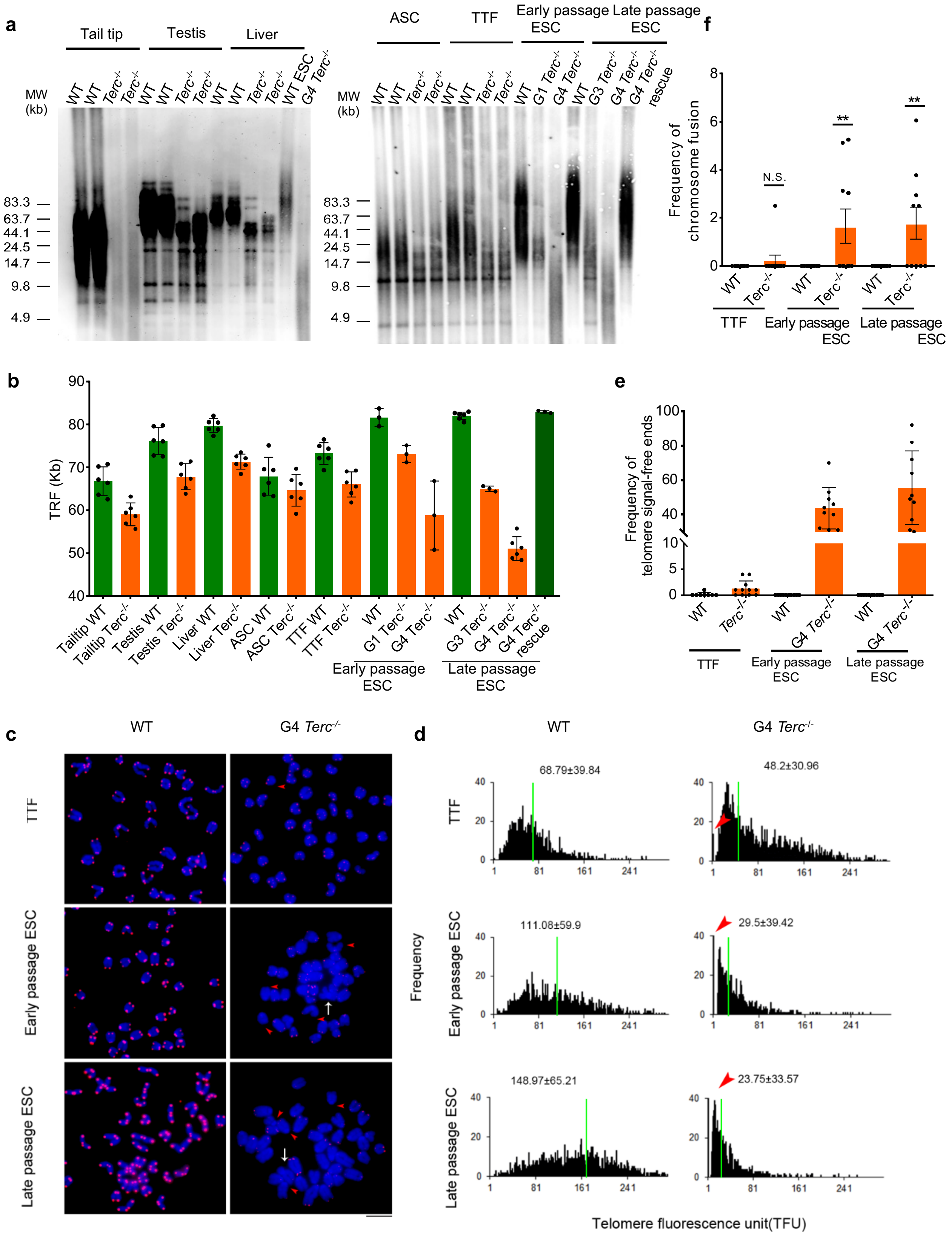

Supplementary Fig. S1. Telomere lengths in wild-type and *Terc*$^{-/-}$ tissues and cells

(a) Telomere restriction fragment (TRF) analysis of telomere length in WT and *Terc*$^{-/-}$ tissues and ESCs. Southern blots were hybridized with a telomere-repeat probe. Early passage G4 *Terc*$^{-/-}$ ESC (passage 18), late passage G4 *Terc*$^{-/-}$ ESC (passage 42), G3 *Terc*$^{-/-}$ TTF (passage 3), G3 *Terc*$^{-/-}$ ASC (passage 3), G1 *Terc*$^{-/-}$ ESC (passage 18) and G3 *Terc*$^{-/-}$ ESC (passage 23) were used in TRF analysis.

(b) Quantitative TRF analysis of telomere length by TeloTool software. Data are shown as mean ± SEM. N ≥ 3 independent experiments.

(c) Representative telomere Q-FISH images of WT and G4 *Terc*$^{-/-}$ cells of early passage (P18) and late passage (P42). Blue, chromosomes stained by DAPI; Red dots, telomeres; White arrows indicate chromosome fusion. Scale bar, 10 μm.

(d) Histogram showing distribution of relative telomere length displayed as TFU by Q-FISH analysis. Green line indicates medium telomere length. Mean ± s.d. of telomere length is shown at the above of each panel.

(e) Frequency of telomere signal-free ends in TTF and early and late passage ESC. Data are shown as mean ± S.E.M.

(f) Frequency of chromosome fusion per metaphase in TTF and early and late passage ESC. Data are shown as mean ± S.E.M.

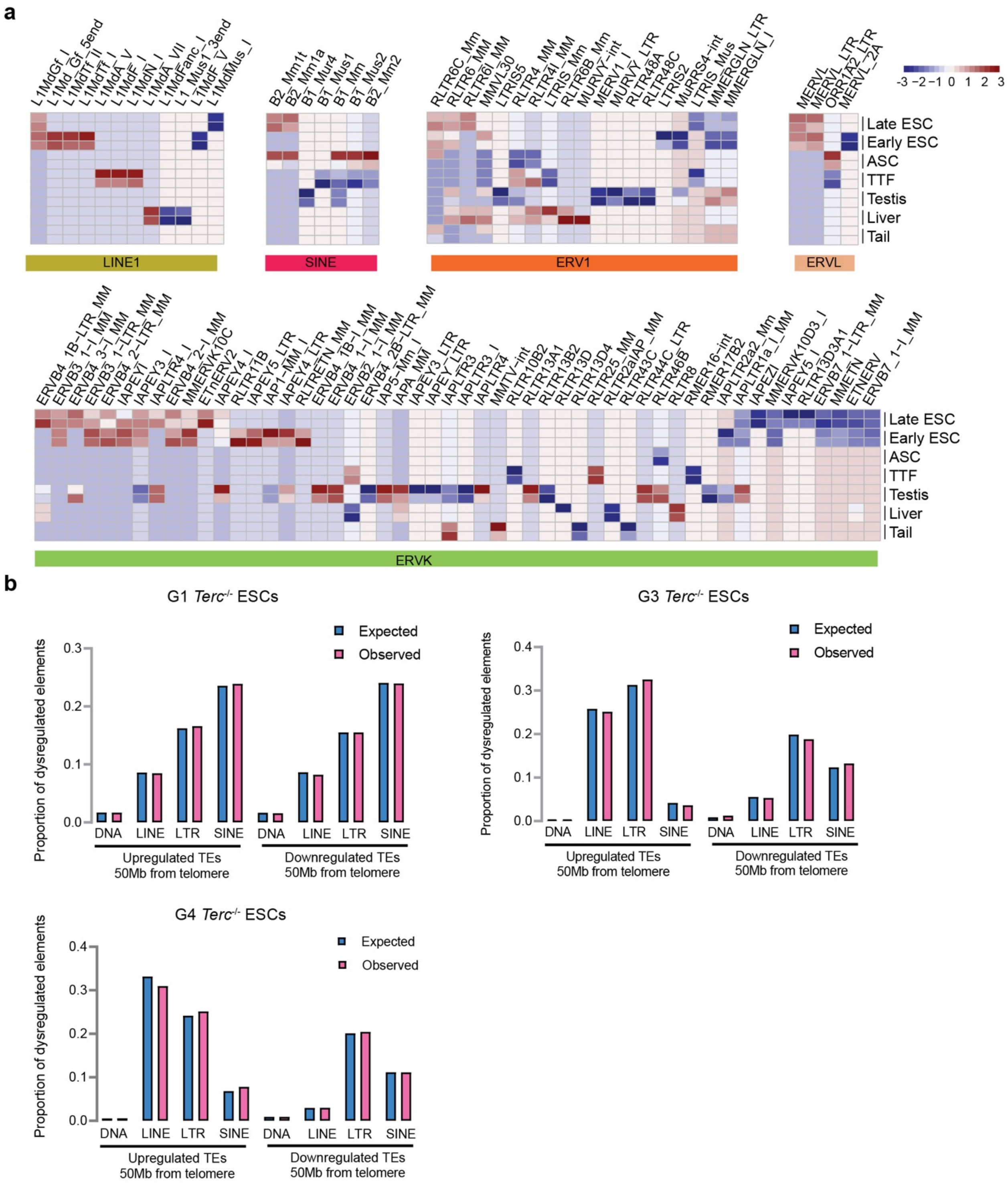

Supplementary Fig. S2. Expression level of differentially expressed transposable elements in wild-type tissues or cells

(a) Expression levels of differentially expressed transposable elements in WT cells; red represents TEs with relatively high expression level in WT cells; blue represents TEs with relatively low expression level in WT cells. The color represents the Z-score value of the TEs.

(b) Enrichment of TEs in G4 *Terc*$^{-/-}$ ESCs located within 50 Mb from the telomere.

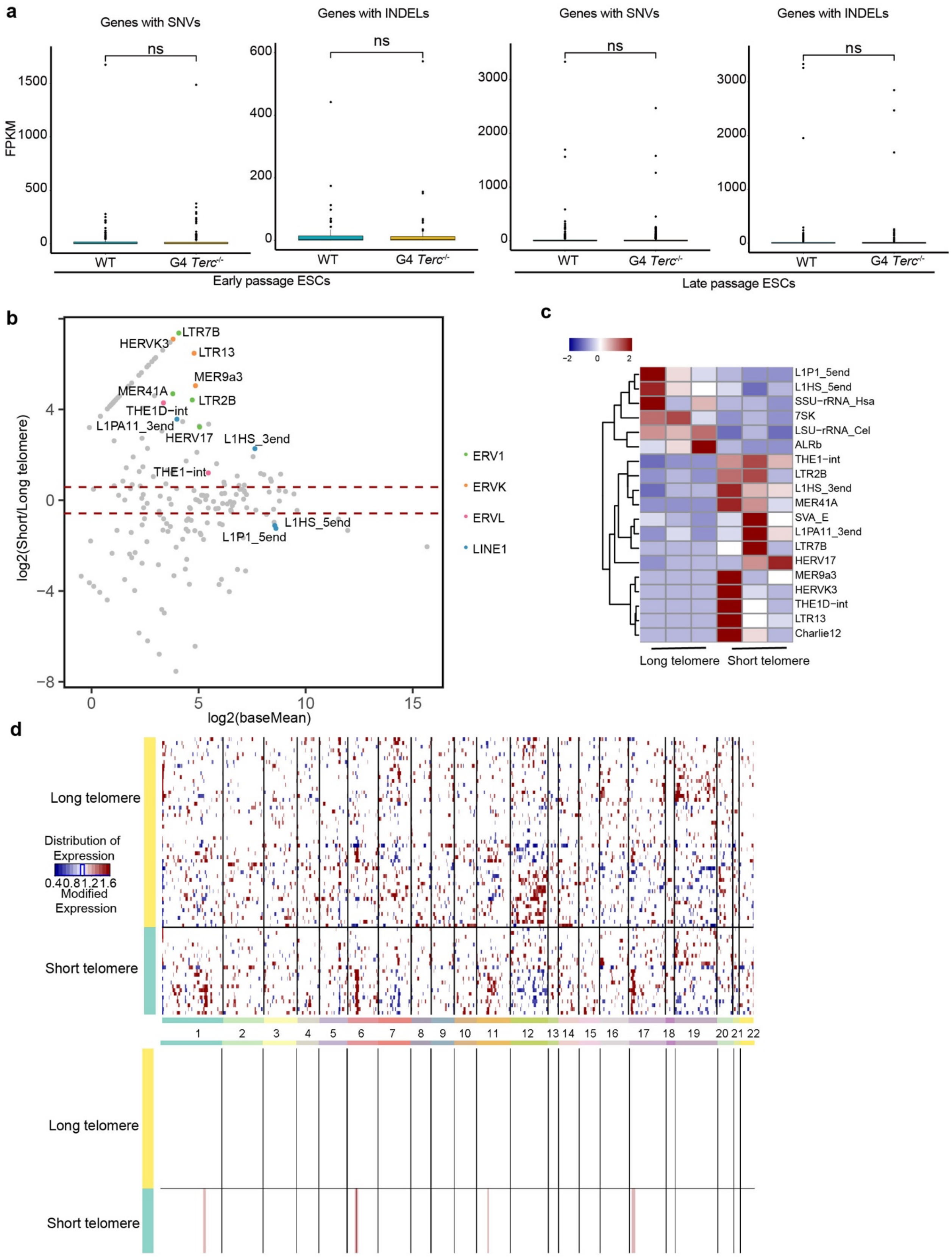
a
Genes with SNVs
Genes with INDELs
Genes with SNVs
Genes with INDELs
ns
FPKM
WT
G4 Terc-/-
Early passage ESCs
Late passage ESCs
b
log2(Short/Long telomere)
log2(baseMean)
LTR7B
HERVK3
LTR13
MER9a3
MER41A
LTR2B
THE1D-int
L1PA11_3end
HERV17
L1HS_3end
THE1-int
L1HS_5end
L1P1_5end
ERV1
ERVK
ERVL
LINE1
c
L1P1_5end
L1HS_5end
SSU-rRNA_Hsa
7SK
LSU-rRNA_Cel
ALRb
THE1-int
LTR2B
L1HS_3end
MER41A
SVA_E
L1PA11_3end
LTR7B
HERV17
MER9a3
HERVK3
THE1D-int
LTR13
Charlie12
Long telomere
Short telomere
d
Long telomere
Distribution of Expression
0.40.81.21.6
Modified Expression
Short telomere
1 2 3 4 5 6 7 8 9 10 11 12 13 14 15 16 17 18 19 20 21 22
Long telomere
Short telomere

Supplementary Fig. S3. Telomere length influences retrotransposons and CNVs in human colorectal cancer cells

(a) The expression levels represented by FPKM of the genes with SNVs/INDELs in early passage ESCs and late passage ESCs, respectively.

(b) Differentially expressed retrotransposons in colorectal cancer (CRC) cells with long or short telomeres. Cancer stem cells have short telomeres whereas cancer epithelial cells show long telomere (Wang et al., 2021).

(c) Heatmap showing differentially expressed retrotransposons. The color bar represents the *Z*-score value of the TEs.

(d) Heatmap of the inferred large-scale CNVs on chromosomal landscape. CNVs distinguished short telomeres from long telomeres for individual cells (rows). The amplifications (red) and deletions (blue) were inferred by averaging the expression of 100 genes stretches on the respective chromosomes (columns). The bottom bars indicate the regions with CNVs in chromosome 1, 6, 11,17.

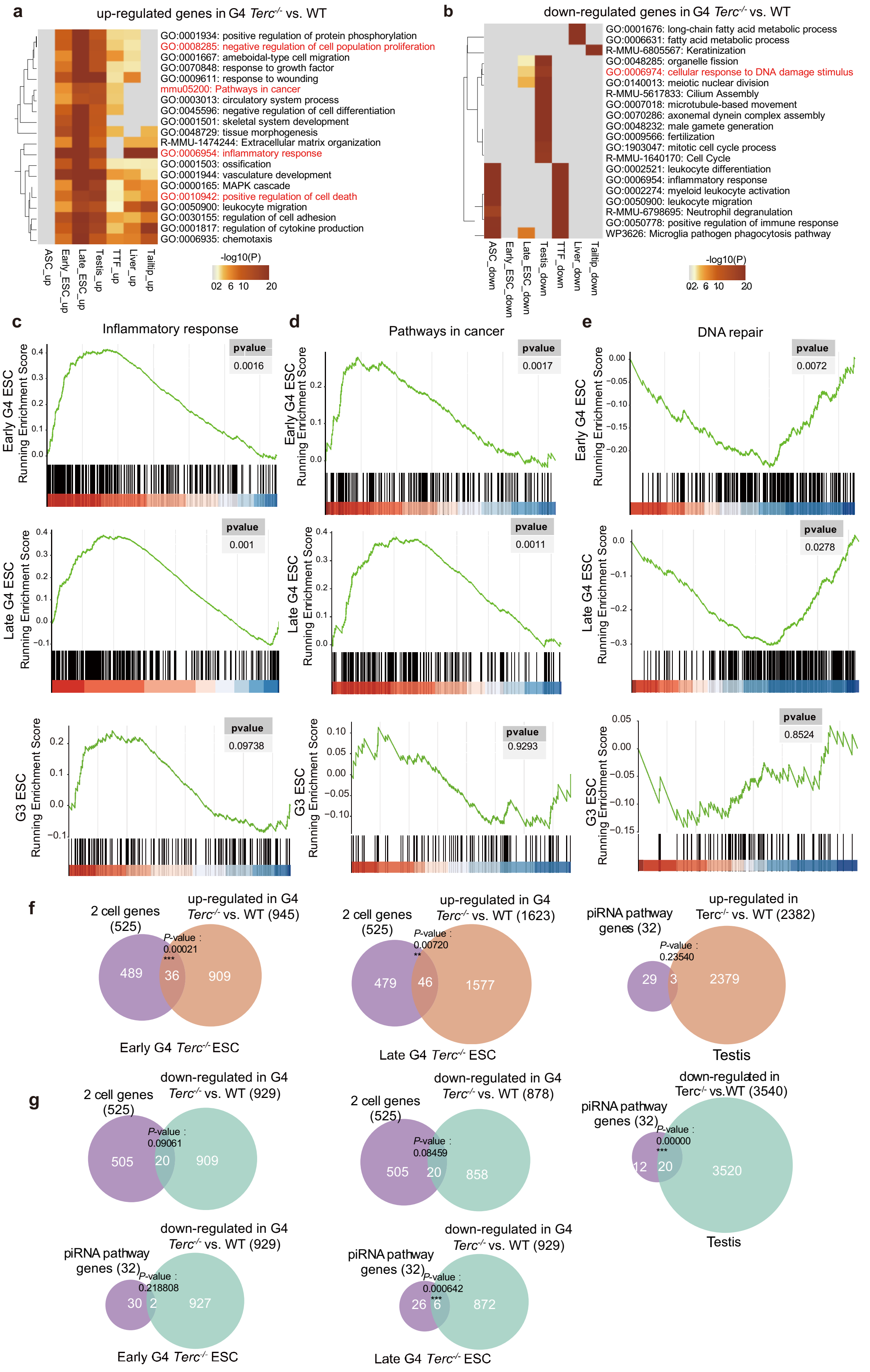

a
up-regulated genes in G4 Terc-/- vs. WT
GO:0001934: positive regulation of protein phosphorylation
GO:0008285: negative regulation of cell population proliferation
GO:0001667: ameboidal-type cell migration
GO:0070848: response to growth factor
GO:0009611: response to wounding
mmu05200: Pathways in cancer
GO:0003013: circulatory system process
GO:0045596: negative regulation of cell differentiation
GO:0001501: skeletal system development
GO:0048729: tissue morphogenesis
R-MMU-1474244: Extracellular matrix organization
GO:0006954: inflammatory response
GO:0001503: ossification
GO:0001944: vasculature development
GO:0000165: MAPK cascade
GO:0010942: positive regulation of cell death
GO:0050900: leukocyte migration
GO:0030155: regulation of cell adhesion
GO:0001817: regulation of cytokine production
GO:0006935: chemotaxis
ASC_up
Early_ESC_up
Late_ESC_up
Testis_up
TTF_up
Liver_up
Tailtip_up
-log10(P)
02 6 10 20
b
down-regulated genes in G4 Terc-/- vs. WT
GO:0001676: long-chain fatty acid metabolic process
GO:0006631: fatty acid metabolic process
R-MMU-6805567: Keratinization
GO:0048285: organelle fission
GO:0006974: cellular response to DNA damage stimulus
GO:0140013: meiotic nuclear division
R-MMU-5617833: Cilium Assembly
GO:0007018: microtubule-based movement
GO:0070286: axonemal dynein complex assembly
GO:0048232: male gamete generation
GO:0009566: fertilization
GO:1903047: mitotic cell cycle process
R-MMU-1640170: Cell Cycle
GO:0002521: leukocyte differentiation
GO:0006954: inflammatory response
GO:0002274: myeloid leukocyte activation
GO:0050900: leukocyte migration
R-MMU-6798695: Neutrophil degranulation
GO:0050778: positive regulation of immune response
WP3626: Microglia pathogen phagocytosis pathway
ASC_down
Early_ESC_down
Late_ESC_down
Testis_down
TTF_down
Liver_down
Tailtip_down
-log10(P)
02 6 10 20
c
Inflammatory response
d
Pathways in cancer
e
DNA repair
Early G4 ESC
Late G4 ESC
G3 ESC
Running Enrichment Score
pvalue
0.0016
0.0017
0.0072
0.001
0.0011
0.0278
0.09738
0.9293
0.8524
f
2 cell genes (525)
up-regulated in G4 Terc-/- vs. WT (945)
P-value : 0.00021 ***
489
36
909
Early G4 Terc-/- ESC
2 cell genes (525)
up-regulated in G4 Terc-/- vs. WT (1623)
P-value : 0.00720 **
479
46
1577
Late G4 Terc-/- ESC
piRNA pathway genes (32)
up-regulated in Terc-/- vs. WT (2382)
P-value : 0.23540
29
3
2379
Testis
g
2 cell genes (525)
down-regulated in G4 Terc-/- vs. WT (929)
P-value : 0.09061
505
20
909
2 cell genes (525)
down-regulated in G4 Terc-/- vs. WT (878)
P-value : 0.08459
505
20
858
piRNA pathway genes (32)
down-regulated in Terc-/- vs.WT (3540)
P-value : 0.00000 ***
12
20
3520
Testis
piRNA pathway genes (32)
down-regulated in G4 Terc-/- vs. WT (929)
P-value : 0.218808
30
2
927
Early G4 Terc-/- ESC
piRNA pathway genes (32)
down-regulated in G4 Terc-/- vs. WT (929)
P-value : 0.000642 ***
26
6
872
Late G4 Terc-/- ESC

Supplementary Fig. S4. Functional analysis of differentially expressed genes by short telomeres

(a-b) Enrichment in the pathways of up-regulated (a) and down-regulated (b) genes in *Terc*$^{-/-}$ vs. WT cells or tissues. The color indicates significance of the enrichment terms. The p-values were calculated through the hypergeometric distribution.

(c-e) The GSEA (Gene Set Enrichment Analysis) enrichment profiles reveal the up- and down-regulated pathways in WT and G4 *Terc*$^{-/-}$ ESCs of early and late passages as well as in G3 *Terc*$^{-/-}$ ESCs. Inflammatory response (c) and cancer-related pathways (d) are enriched among upregulated genes. DNA repair pathway (response to DNA damage) is enriched within downregulated genes (e). Red bars at the bottom show the genes up-regulated in *Terc*$^{-/-}$ vs. WT ESCs, whereas blue bars represent the genes down-regulated in *Terc*$^{-/-}$ vs. WT ESCs.

(f) Venn diagram displaying the overlaps between 2-cell genes and up-regulated genes after *Terc*$^{-/-}$ knocking out in ESCs, and the overlaps between piRNA pathway genes and up-regulated genes after *Terc*$^{-/-}$ knocking out in testis. The *p*-value was calculated with the hypergeometric distribution method.

(g) Venn diagram displaying the overlaps between 2-cell genes/piRNA pathway genes and down-regulated genes after *Terc*$^{-/-}$ knocking out in ESCs, and the overlaps between piRNA pathway genes and down-regulated genes after *Terc*$^{-/-}$ knocking out in testis. The *p*-value was calculated with the hypergeometric distribution method.

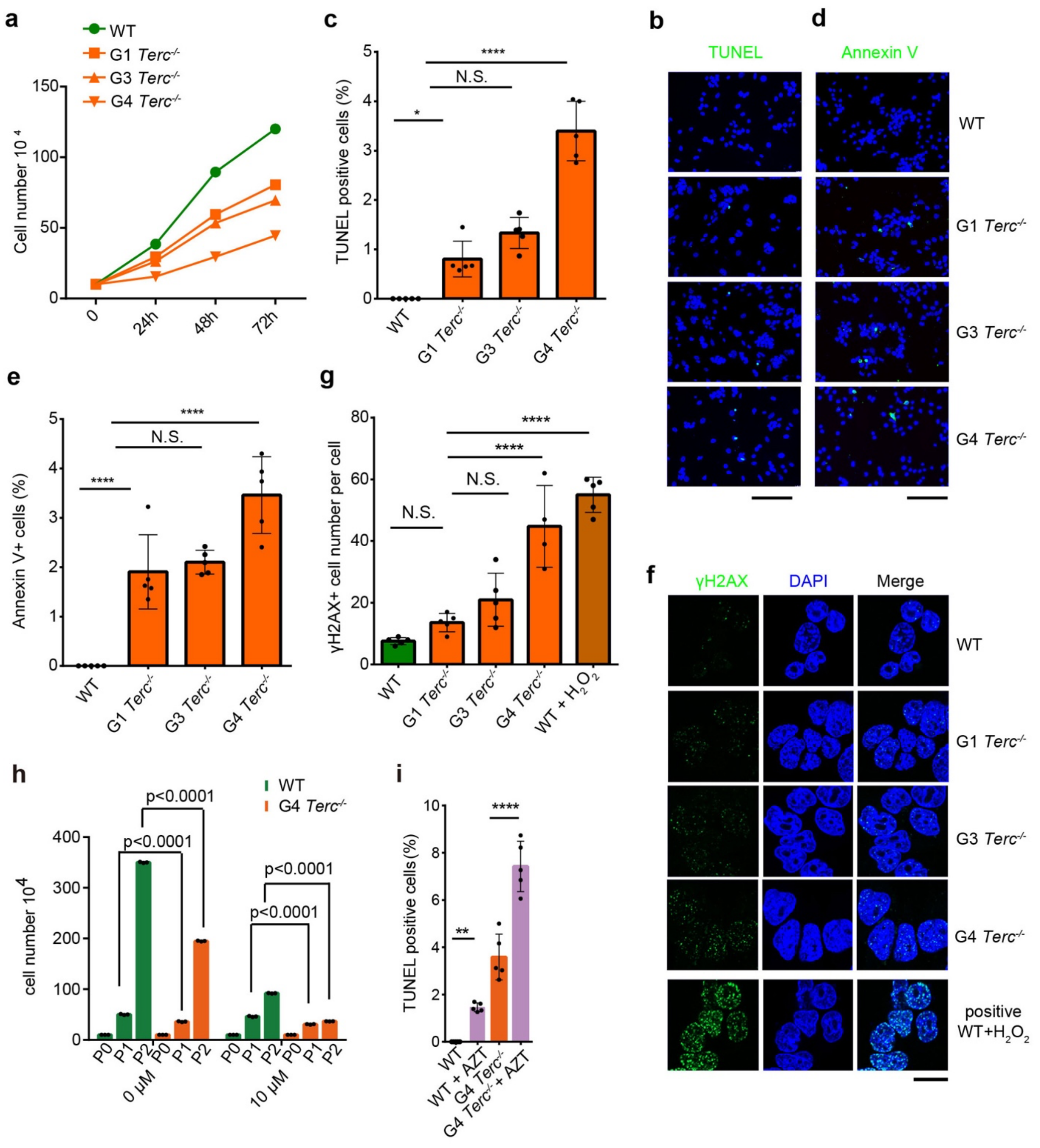

Supplementary Fig. S5. Telomere shortening affects ESC proliferation and causes DNA damage

(a) Growth curve of WT, G1 *Terc*$^{-/-}$, G3 *Terc*$^{-/-}$ and G4 *Terc*$^{-/-}$ ESCs.

(b) TUNEL assay of apoptosis cells in WT, G1 *Terc*$^{-/-}$, G3 *Terc*$^{-/-}$ and G4 *Terc*$^{-/-}$ ESCs. Scale bar, 10 μm.

(c) Quantification of TUNEL positive cells in ESCs.

(d) Early stages of cell death visualized by the Annexin V assay in WT, G1 *Terc*$^{-/-}$, G3 *Terc*$^{-/-}$ and G4 *Terc*$^{-/-}$ ESCs. Scale bar, 10 μm.

(e) Quantification of Annexin V-positive cells in ESCs.

(f) Immunofluorescence of γH2AX (green) of WT, G1 *Terc*$^{-/-}$, G3 *Terc*$^{-/-}$ and G4 *Terc*$^{-/-}$ ESCs. Scale bar, 10 μm.

(g) Statistical analysis of γH2AX foci per cell in ESCs. Scale bar, 10 μm. Error bars indicate mean ± SD. Ns, non-significant; *, $p < 0.05$; ****, $p < 0.0001$.

(h) Number of WT and G4 *Terc*$^{-/-}$ ESCs in cultures containing DMSO (control, AZT 0 μM) or Zidovudine (AZT 10 μM). Cells were counted and re-plated every passage.

(i) TUNEL assay of apoptosis in WT and *Terc*$^{-/-}$ ESCs before and after treatment with AZT. Statistical analysis was determined by student *t*-test, ** means $p < 0.01$, **** means $p < 0.0001$.

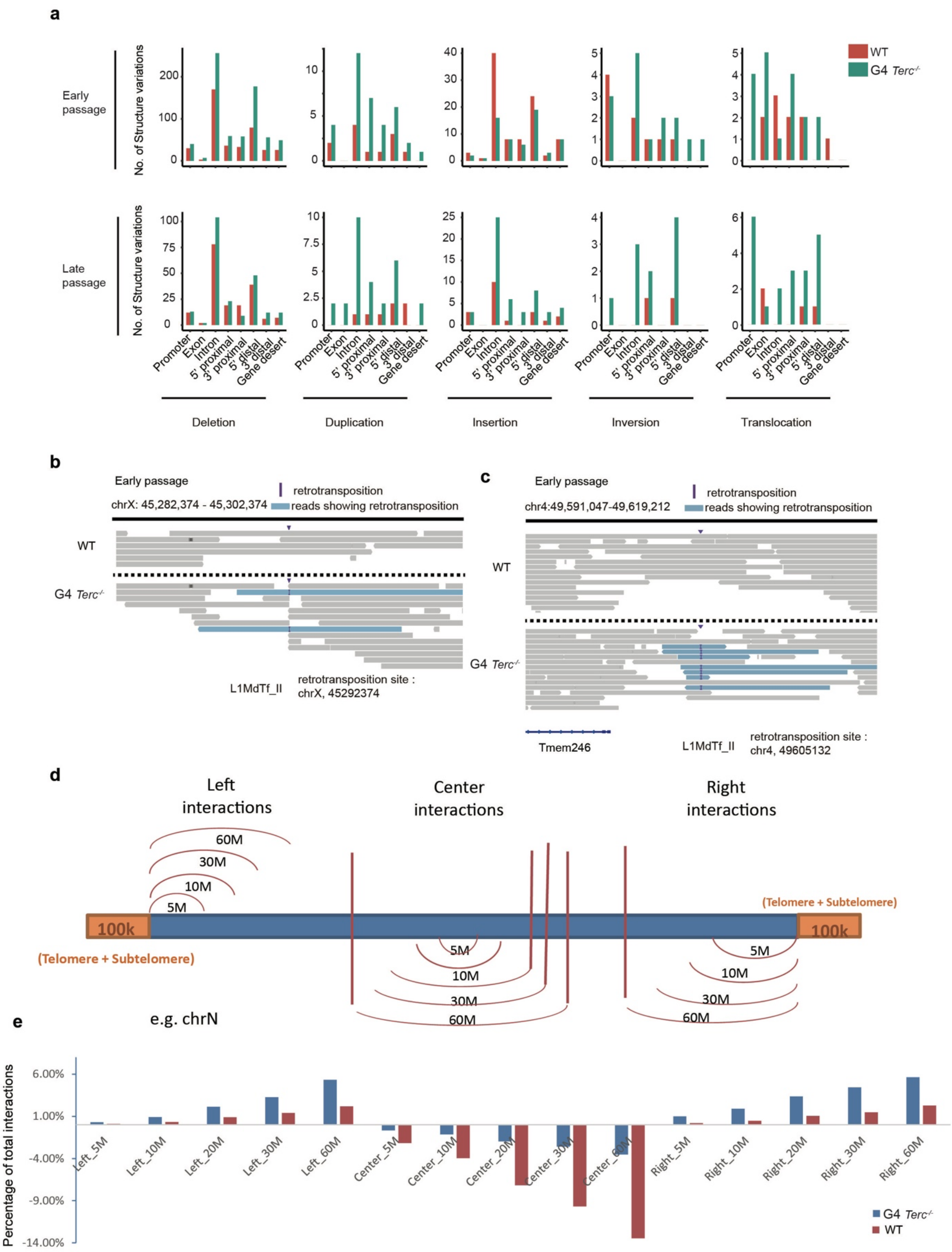

Supplementary Fig. S6. Distribution of mutation events

(a) Bar plot showing the proportion of chromatin structure variations occurred on the genome.

Promoter, 2 kb around transcriptional start sites; 5′ proximal, 2–10 kb upstream of gene; 5′ distal, 10–100 kb upstream of gene; 3′ proximal, 0–10 kb downstream of gene; 3′ distal, 10–100 kb downstream of gene; Gene desert, > 100 kb away from the nearest gene in wild-type and G4 *Terc*$^{-/-}$ ESCs at early or late passage.

(b-c) Two examples for the location of L1MdTf_II insertion sequencing tags in wild-type and G4 *Terc*$^{-/-}$ ESCs. The reads supporting the insertion are marked as blue and the insertion sites are marked as “I”.

(d) Schematic describing the regions used in calculating interactions of sub-telomeric regions and center region of chromosome.

(e) Percentage interactions at different chromatin regions in G4 *Terc*$^{-/-}$ ESCs after minus that in WT ESCs.

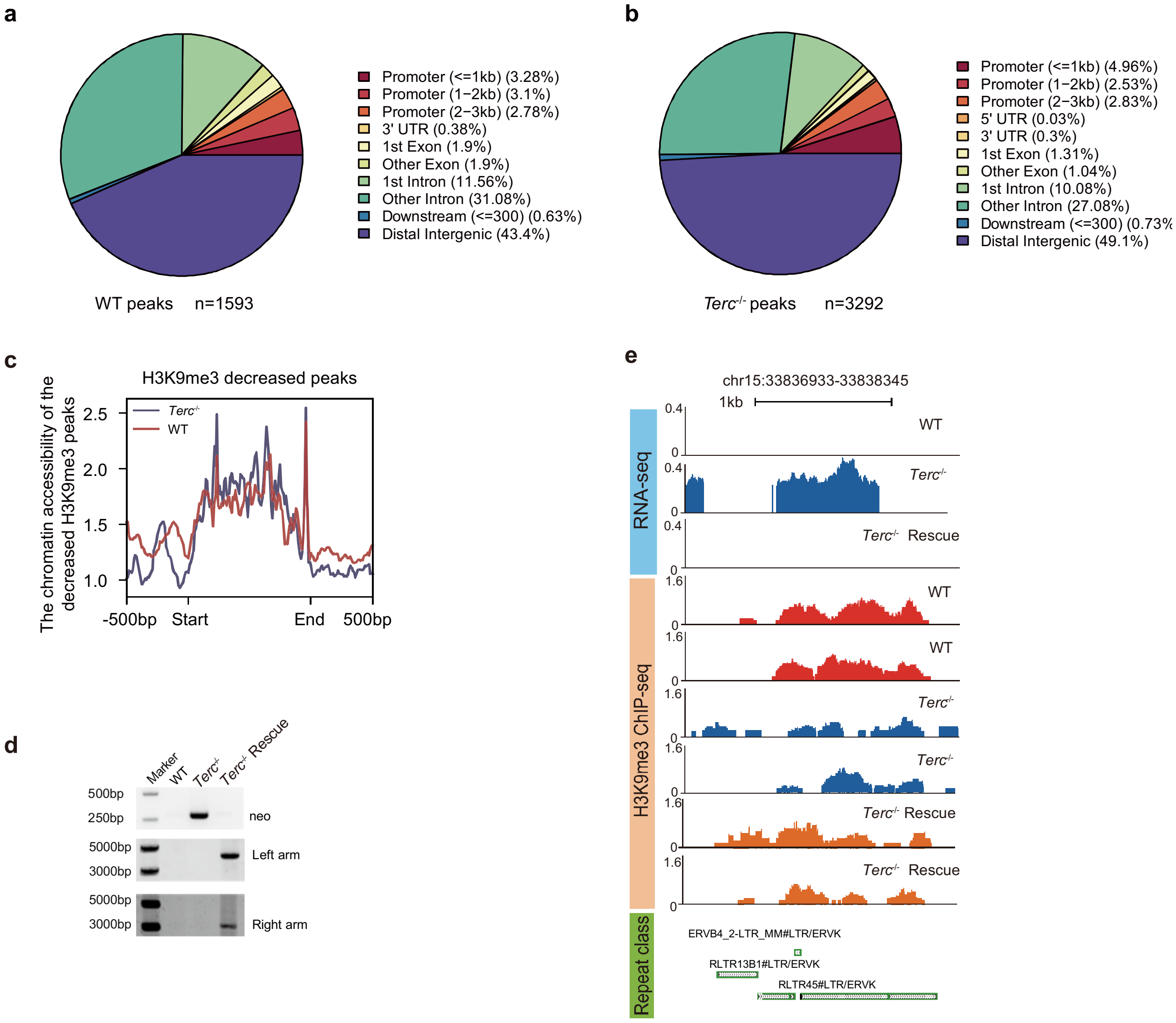

Supplementary Fig. S7. Open chromatin with reduced H3K9me3 deposition after telomere shortening

(a-b) The peak of ATAC-Seq on the genome regions including the promoter, 3' UTR, 5' UTR, 1st exon, other exon, 1st intron, other intron, downstream region and the distal intergenic regions in WT (a) and G4 *Terc*$^{-/-}$ (b) ESCs.

(c) The chromatin accessibility in the regions with decreased H3K9me3 enrichment.

(d) PCR analysis of *Terc* knockin. PCR results revealed that the Neo gene was only detected in G4 *Terc*$^{-/-}$ ESCs, while both left and right homology arm used in *Terc* knock-in were detected in *Terc*-rescued ESCs.

(e) The UCSC profile showing the signal of the RNA-Seq and H3K9me3 ChIP-Seq around the retrotransposon ERVB4_2-LTR_MM locus in WT, G4 *Terc*$^{-/-}$ and G4 *Terc*-rescue ESCs. Y-axis represents relative RPKM of sequencing reads. Repeat classes are indicated at bottom of the figure in green-edged boxes.

Supplementary Table S1. Primers for T/S ratio by qPCR and *Terc* repair

mTel F	CGGTTTGTTTGGGTTTGGGTTTGGGTTTGGGT TTGGGTT
mTel R	GGCTTGCCTTACCCTTACCCTTACCCTTACCCT TACCCT
36B4 F	ACTGGTCTAGGACCCGAGAAG
36B4 R	TCAATGGTGCCTCTGGAGATT
Repair-Homolog-L-F	GGTACCCCCTACTTCATCTCTGTTGTTGGA
Repair-Homolog-L-R	CTCGAGGGTGTCTTGTGTCTCTGTCTACAT
Repair-Terc-F	GTCGACTCCCGATGCCCTCTATTGTTTTTA
Repair-Terc-R	GAATTCCACAAACATAGTTACCCCTGAGGA
Repair-Homolog-R-F	CCCGGGCAAGATTCTGAAACCCAACGTCAA
Repair-Homolog-R-R	GCGGCCGCTTCACACTACAGTCTAAGCTGTCC
Terc-Repair-Cas9-L-Oligo-F	CACCGCTGATAGTGCTAACACTGC
Terc-Repair-Cas9-L-Oligo-R	AAACGCAGTGTTAGCACTATCAGC
Terc-Repair-Cas9-R-Oligo-F	CACCGCAGCTGTGCCTTGAGATCCG
Terc-Repair-Cas9-R-Oligo-R	AAACCGGATCTCAAGGCACAGCTGC
Repair-Genotype-Homolog-L-F F1	AGATGTTTGACAGGTGGGTAAGA
Repair-Genotype-Homolog-L-F R1	CCTACCGGTGGATGTGGAATGT
Repair-Genotype-Homolog-L-F F2	AACCTCCCCTTCTACGAGCG
Repair-Genotype-Homolog-L-F R2	CTTCACCTTTGAGAGATAGAGCCA
Genotype-neo-F	CTTGGGTGGAGAGGCTATTC
Genotype-neo-R	AGGTGAGATGACAGGAGATC
Genotype-Terc-F	CATTAGCTGTGGGTTCTGGTCT
Genotype-Terc-R	TCCTGCGCTGACGTTTGTTT
